# Hijacking antibody-induced CTLA-4 lysosomal degradation for safer and more effective cancer immunotherapy

**DOI:** 10.1038/s41422-019-0184-1

**Published:** 2019-07-02

**Authors:** Yan Zhang, Xuexiang Du, Mingyue Liu, Fei Tang, Peng Zhang, Chunxia Ai, James K. Fields, Eric J. Sundberg, Olga S. Latinovic, Martin Devenport, Pan Zheng, Yang Liu

**Affiliations:** 1 Divisions of Immunotherapy, University of Maryland Baltimore School of Medicine, Baltimore, MD 21201 USA; 2 Divisions of Basic Science Division, Institute of Human Virology, University of Maryland Baltimore School of Medicine, Baltimore, MD 21201 USA; 3 Graduate Program in Molecular Microbiology and Immunology, University of Maryland Baltimore School of Medicine, Baltimore, MD 21201 USA; 4 Department of Microbiology and Immunology, University of Maryland Baltimore School of Medicine, Baltimore, MD 21201 USA; 5 OncoImmune, Inc, Rockville, MD 20850 USA; 6 Department of Surgery, University of Maryland Baltimore School of Medicine, Baltimore, MD 21201 USA

**Keywords:** Tumour immunology, Cancer immunotherapy

## Abstract

It remains unclear why the clinically used anti-CTLA-4 antibodies, popularly called checkpoint inhibitors, have severe immunotherapy-related adverse effects (irAEs) and yet suboptimal cancer immunotherapeutic effects (CITE). Here we report that while irAE-prone Ipilimumab and TremeIgG1 rapidly direct cell surface CTLA-4 for lysosomal degradation, the non-irAE-prone antibodies we generated, HL12 or HL32, dissociate from CTLA-4 after endocytosis and allow CTLA-4 recycling to cell surface by the LRBA-dependent mechanism. Disrupting CTLA-4 recycling results in robust CTLA-4 downregulation by all anti-CTLA-4 antibodies and confers toxicity to a non-irAE-prone anti-CTLA-4 mAb. Conversely, increasing the pH sensitivity of TremeIgG1 by introducing designed tyrosine-to-histidine mutations prevents antibody-triggered lysosomal CTLA-4 downregulation and dramatically attenuates irAE. Surprisingly, by avoiding CTLA-4 downregulation and due to their increased bioavailability, pH-sensitive anti-CTLA-4 antibodies are more effective in intratumor regulatory T-cell depletion and rejection of large established tumors. Our data establish a new paradigm for cancer research that allows for abrogating irAE while increasing CITE of anti-CTLA-4 antibodies.

## Introduction

CTLA-4 interacts with CD80 and CD86^1–3^ to ensure proper function of regulatory T cells^4^ and protect host against autoinflammatory diseases.^5–8^ Anti-CTLA-4 monoclonal antibodies (mAbs) have demonstrated strong and broad cancer immunotherapeutic effects (CITE) in a variety of preclinical models^9–11^ and are used clinically both as monotherapy^12,13^ and as part of combination therapy with Nivolumab.^14,15^ However, compared with anti-PD-1/PD-L1 antibodies, CTLA-4-targeting in cancer patients has been less successful. Head-to-head comparisons have revealed that response rates of melanoma patients to the anti-CTLA-4 antibody, Ipilimumab, was consistently lower than for an anti-PD-1 antibody.^15–17^ Thus, while anti-PD-1/PD-L1 antibodies have gained approval for clinical use in rapidly expanding indications, monotherapy with anti-CTLA-4 antibodies have failed in multiple phase III clinical trials apart from melanoma.^18^ Moreover, CTLA-4 monotherapy has more immunotherapy-related adverse effects (irAEs) than anti-PD-1/PD-L1 therapy.^19^ In addition, the rate of severe irAE (Grades 3 and 4) reached 73–90% in neo-adjuvant therapy of melanoma patients receiving combination of Ipilimumab and Nivolumab.^20,21^ The strong irAEs further limit the doses tolerated by cancer patients. Nevertheless, combination with anti-PD-1 resulted in significantly improved response rates and patient survival in multiple types of cancer.^14,15,17,22–24^ Furthermore, anti-CTLA-4 antibodies are capable of inducing long-lasting immunity in cancer patients.^25,26^ Therefore, CTLA-4 remains an important immunotherapy target, but major challenges remain in improving both safety and efficacy of anti-CTLA-4 mAbs.

In order to generate safer and more effective anti-CTLA-4 antibodies, it is critical to understand the molecular basis underlying irAE and CITE of anti-CTLA-4 antibodies. Traditionally viewed as checkpoint inhibitors, anti-CTLA-4 antibodies have been postulated to achieve immunotherapy by antagonizing the endogenous function of CTLA-4.^27,28^ Since genetic inactivation of CTLA-4 in mice and humans has caused severe autoimmune diseases,^5–7^ an effective antagonist of CTLA-4 molecule is likely to induce autoimmune diseases, making irAE a necessary price for cancer immunity. In this context, we have recently reported that blocking the interaction between CTLA-4 and its ligands CD80 and CD86 is neither necessary nor sufficient for CITE of anti-CTLA-4 antibodies.^29^ In contrast, studies from several laboratories, including ours, established that selective depletion of regulatory T cells in the tumor microenvironment (TME) but not in the normal tissues as the primary mechanism of action of CITE.^29–33^ The new understanding of regulatory T cell depletion explained why it is possible to uncouple irAE from CITE.^33,34^

Unlike most cell-surface molecules, CTLA-4 recycles between the cell surface and endosomes,^35^ where it is prevented from lysosomal degradation and recycles back to the cell surface by binding to the lipopolysaccharide-responsive and beige-like anchor (LRBA) protein.^8,36^ Since genetic mutations in either *CTLA-4*^5,8^ or *LRBA*^8,36^ cause autoimmune diseases in human, we hypothesize that anti-CTLA-4-induced irAE may relate to antibody-mediated disruption of CTLA-4 recycling. Here we systemically tested this hypothesis and report that disruption of CTLA-4 recycling underlies both irAE and suboptimal tumor rejection of clinical anti-CTLA-4 antibodies. In contrast, anti-CTLA-4 antibodies that dissociate from CTLA-4 in endosomes allow normal recycling of both antibodies and CTLA-4 and exhibit dramatically less irAE but improved immunotherapeutic effect. Our work provides a new paradigm in the field on how to target CTLA-4 effectively for cancer immunotherapy.

## Results

### Ipilimumab markedly downregulates the level of cell surface CTLA-4

**Fig. 1:**
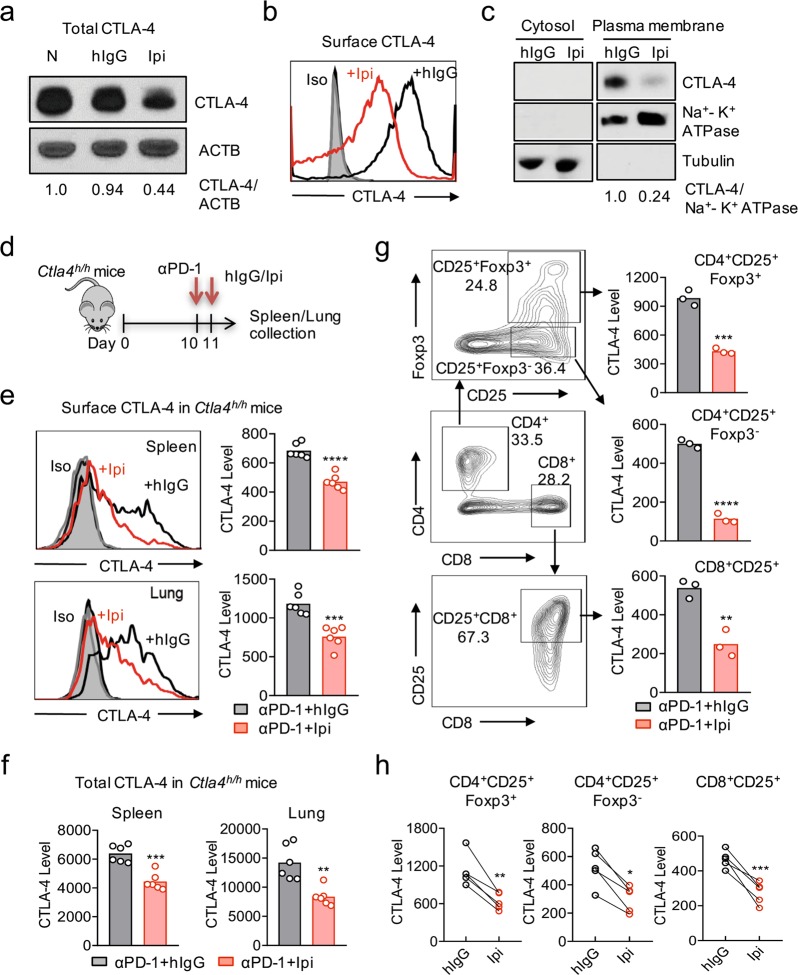
Ipilimumab downregulates cell CTLA-4. **a** CHO cells stably expressing human CTLA-4 were treated with either control human IgG or Ipilimumab (Ipi) for 4 h at 37 °C, then the CTLA-4 protein levels were analyzed by western blot. **b** Cell surface CTLA-4 on CHO cells used in (**a**) was measured by flow cytometry. To avoid the caveat associated with CTLA-4 masking by residual antibodies from the treatment phase, the cells were incubated with Ipilimumab (10 μg/mL) for 30 min at 4 °C before the staining with the non-competing anti-CTLA-4 antibody, clone BNI3. **c** Plasma membrane and cytosolic proteins purified from (**b**) were detected for CTLA-4 by immunoblot. Na^+^-K^+^ ATPase and Tubulin were used as controls for purity of cellular fractionation. **d** Ten-days old *Ctla4*^*h/h*^ mice (body weight: 4.5–5.3 g; *n* = 6) were treated with anti-mouse PD-1 antibody intraperitoneally (i.p.) (100 μg/mouse). 24 h later, mice were further treated (i.p.) with 100 μg of control hIgG Fc or Ipilimumab for 4 h. **e**, **f** Cell surface (**e**) and total CTLA-4 (**f**) in Tregs isolated from spleen or lung were evaluated by flow cytometry. **g**, **h** Human PBMCs from healthy donors were stimulated with anti-CD3/anti-CD28 for 2 days and then treated with either control hIgGFc or Ipilimumab for 4 h at 37 °C. Cell surface CTLA-4 on CD4^+^CD25^+^FOXP3^+^ Tregs, CD4^+^CD25^+^FOXP3^-^ non-Tregs and CD8^+^CD25^+^ cells were measured by flow cytometry. Again, for all the flow cytometry data of CTLA-4-positive cells treated with Ipilimumab in vitro or in vivo, stainings were performed in the presence of excess Ipilimumab to exclude bias associated with Ipilimumab masking of BNI3. Data are mean ± SEM. *p < 0.05, **p < 0.01, ***p < 0.001, ****p < 0.0001. Data in (**a**–f) have been reproduced in three independent experiments. Data in (**g**) and (**h**) are a representative of 5 human samples, with the summary of them presented in (**h**)

We evaluated the impact of irAE-prone anti-CTLA-4 antibody Ipilimumab in cells with ectopic expression of CTLA-4 by both immunoblot and by flow cytometry. A potential caveat in measuring antibody-induced CTLA-4 downregulation is CTLA-4 masking by pre-existing antibodies, although no such caveats exist for immunoblot in which the antibody–antigen complex would be disrupted during SDS–PAGE. To overcome this caveat, we identified an anti-CTLA-4 antibody (clone BNI3) that has minimal cross-blocking with Ipilimumab (Supplementary Information, Fig. S1a). Any residual masking of cell surface CTLA-4 by Ipilimumab is normalized by performing staining in the presence of excess Ipilimumab. Immunoblot demonstrated that Ipilimumab markedly reduced total CTLA-4 in CHO cells stably expressing CTLA-4 (Fig. 1a). A similar effect was observed when total CTLA-4 molecules in transient CTLA-4 transfectants of 293T cells were assessed by flow cytometry (Supplementary Information, Fig. S1b). As shown in Fig. 1b, cell surface CTLA-4 is reduced by approximately 10-fold after a short-term treatment of Ipilimumab. This is substantiated by immunoblot detection of CTLA-4 in plasma membrane (Fig. 1c). Similar results were observed in 293T cells (Supplementary Information, Fig. S1c).

To test if Ipilimumab-triggered CTLA-4 downregulation is relevant for its irAE, we analyzed the CTLA-4 levels on Tregs from young human *CTLA4* gene knockin mice (*Ctla4*^*h/h*^),^34^ which we have shown are highly susceptible to irAE, especially those that received anti-PD-1/Ipilimumab combination therapy.^33^ Since anti-PD-1 treatment increased cell surface CTLA-4 (Supplementary Information, Fig. S1d, e), we tested the effect of Ipilimumab in mice that received anti-PD-1 antibody 24 h prior to Ipilimumab treatment (Fig. 1d). As shown in Fig. 1e, f, at 4 h after Ipilimumab treatment, significant downregulation of both cell surface (Fig. 1e) and total (Fig. 1f) CTLA-4 was observed in Tregs from lung and spleen. Likewise, tumor-bearing mice also expressed higher levels of cell surface CTLA-4 than naïve mice, allowing us to reveal downregulation of CTLA-4 by Ipilimumab in the absence of anti-PD-1 treatment (Supplementary Information, Fig. S1f, g). To test if Ipilimumab downregulates CTLA-4 in human T cells, we incubated activated human T cells with Ipilimumab for 4 h and evaluated CTLA-4 levels in T cells by flow cytometry. As shown in Fig. 1g, downregulation of surface CTLA-4 was observed in both activated FOXP3^+^/FOXP3^−^ CD4 T cells and activated CD8^+^ cells. Similar results were observed in 5 different human blood samples tested (Fig. 1g, h).

### Downregulation of surface CTLA-4 by anti-CTLA-4 mAbs correlates with their irAE

Our previous study showed that different anti-human CTLA-4 show drastically different irAE.^33^ Thus, while Ipilimumab+anti-PD-1 treatment induces severe growth retardation, death, anemia and multi-organ inflammation, anti-PD-1 combination with HL12 or HL32 caused no growth retardation, anemia nor death, and minimal leukocyte infiltration to organs.^33^ Since Tremelimumab was also found to have strong irAE in the clinic,^37^ we generated an IgG1 variant of Tremelimumab (TremeIgG1) to enable evaluation of CITE along with irAE in vivo. We found it has much stronger irAE than HL12 in the mouse irAE model (Supplementary Information, Fig. S2a–d) and potent CITE (Supplementary Information, Fig. S2e).

**Fig. 2:**
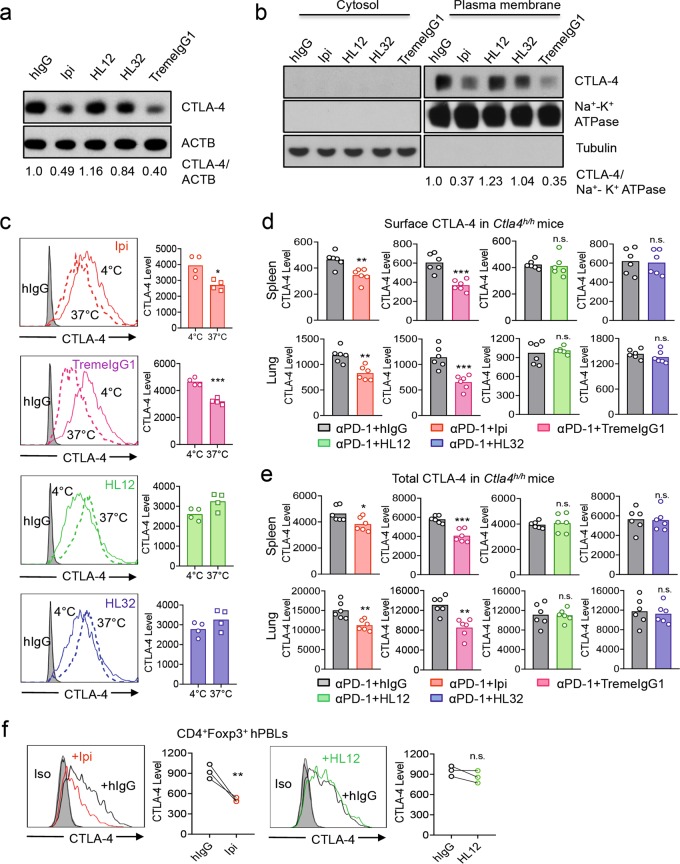
Downregulation of CTLA-4 by anti-CTLA-4 mAbs correlates with their irAE. **a** Human embryonic kidney (HEK) 293T cells were transiently transfected with *CTLA4* cDNA and were incubated with indicated control hIgGFc, Ipilimumab, TremeIgG1, HL12 and HL32, respectively, for 4 h. The CTLA-4 protein level was analyzed by western blot. ACTB was used as loading control. **b** As in (**a**) and cytosolic and plasma membrane fractions were isolated and tested for CTLA-4 protein levels by immunoblot, and that the Tubulin and Na^+^-K^+^ ATPase were used as loading and purity controls for cellular fractionation. **c** CHO stable cell lines expressing hCTLA-4 were treated with Ipilimumab, TremeIgG1, HL12 or HL32 at 4 °C for 30 min. Half of the cells were kept at 4 °C (solid lines), and the other half were switched to 37 °C for another 2 h (dashed lines). After washing out unbound antibodies at 4 °C, cell surface CTLA-4 was detected by an AF488-conjugated anti-human Fc antibody at 4 °C and analyzed by flow cytometry. The representative histograms are shown on the left, and summary data are shown in the right. **d**, **e** Ten-day old *Ctla4*^*h/h*^ mice (body weight: 4.5–5.3 g; *n* = 6) were treated with anti-PD-1 (i.p.100 μg/mouse). 24 h later, mice were treated with 100 μg of control hIgGFc, Ipilimumab, TremeIgG1, HL12, or HL 32. 4 h later, cell surface (**d**) and total (**e**) CTLA-4 of spleen and lung Tregs were evaluated by flow cytometry. To avoid the caveat associated with CTLA-4 masking by residual antibodies from in vivo treatment, saturating doses of Ipilimumab, TremeIgG1, HL12 or HL32 were added before CTLA-4 staining by BNI3 clone when comparing with hIgG group. **f** Human PBMCs from healthy donors’ blood were stimulated by anti-CD3/anti-CD28 for 2 days and treated with either control hIgG, Ipilimumab or HL12 for 4 h at 37 °C. Surface CTLA-4 of CD4^+^FOXP3^+^ Tregs was measured by flow cytometry. Data are mean ± SEM. *p < 0.05, **p < 0.01, ***p < 0.001, ****p < 0.0001. Unpaired two-tailed Student’s t-test. Representative data of three independent experiments in (**a**) and (**b**) were shown. Summary data from two independent experiments (two samples per group) are shown in (**c**). Data in (**d**) and (**e**) have been repeated at least three times in both male and female mice. Data from 3 healthy donors are shown in (**f**). Again, for all the flow cytometry data of CTLA-4-positive cells treated with anti-CTLA-4 mAbs in vitro or in vivo, stainings were performed in the presence of excess treated antibodies to exclude possible masking of BNI3 epitope by antibodies used during the treatment phase

With two irAE-prone and two non-irAE-prone human IgG1 anti-CTLA-4 antibodies, we tested whether CTLA-4 downregulation correlates with irAE. We first compared these antibodies for their dowregulation of total and plasma membrane-associated CTLA-4 by Immunoblot as this test is unaffected by antibody masking. As shown in Fig. 2a, at 4 h after treatment, both irAE-prone antibodies (Ipilimumab and TremeIgG1) reduced total CTLA-4 in CTLA-4-transfected 293T cells. While CTLA-4 accumulation in plasma membrane was reduced selectively by irAE-prone anti-CTLA-4 antibodies, non-irAE-prone antibodies (HL12 and HL32) had no effect on either total or plasma membrane-associated anti-CTLA-4 antibodies (Fig. 2b). Since CTLA-4 is associated with plasma and organelle membrane, it was not present in the cytosol (Fig. 2b).

The effects of these anti-CTLA-4 mAbs on surface CTLA-4 were also tested by flow cytometry. We incubated CHO cells stably expressing CTLA-4 with both irAE-prone and non-irAE-prone anti-CTLA-4 antibodies either at 4 °C (no downregulation) or 37 °C. The amounts of cell surface antibody-bound CTLA-4 molecules were measured using an Alexa-Fluor 488 (AF488)-conjugated anti-human IgG Fc secondary antibody. Despite the potential arrival of new CTLA-4 molecules, less CTLA-4 was found on the surface of cells that were treated with either Ipilimumab or TremeIgG1. Thus, both Ipilimumab and TremeIgG1 decreased cell surface CTLA-4 when incubated with CTLA-4-expressing CHO cells. In contrast, HL12 and HL32 caused no reduction of surface CTLA-4 (Fig. 2c). A slight increase of cell surface CTLA-4 was observed in the HL12- and HL32-treated cells at 37 °C, which can be attributed to newly synthesized CTLA-4. Thus, our data in CHO cell demonstrated a strong correlation between CTLA-4 downregulation and irAE.

To substantiate this observation in T cells, we compared the effect of different antibodies on CTLA-4 expression in *Ctla4*^*h/h*^ mice.^34^ To avoid the antibody masking, we first tested whether CTLA-4 staining antibody (BNI3 clone) has binding competition with TremeIgG1, HL12 or HL32 on T cells. We incubated human primary peripheral blood mononuclear cells (PBMCs) with either hIgG or anti-CTLA-4 mAbs at 4 °C before BNI3 staining, and compared the change of CTLA-4 level. As shown in Supplementary Information, Fig. S2f, Ipilimumab and TremeIgG1 had no effect on BNI3 binding, although HL12 and HL32 appeared to have slight effect on the CTLA-4 staining by BNI3 clone. To normalize any effect associated with antibody masking, we have added excess amount of anti-CTLA-4 antibodies in the staining step. We focused on setting in which anti-CTLA-4 was used in combination with anti-PD-1 in vivo, as this condition caused most severe and frequent irAE in the clinic and in our model. As shown in Fig. 2d, e, both Ipilimumab and TremeIgG1 downregulated cell-surface and total CTLA-4 level in Tregs from spleen and lung. In contrast, HL12 and HL32 had no effect on CTLA-4 level of Tregs in the same model. To confirm the impact of these antibodies in human Treg, we also compared the effect of the four antibodies on activated human T cells. As shown in Fig. 2f, significant reduction of CTLA-4 was induced by Ipilimumab but not by HL12 in human CD4^+^FOXP3^+^ T cells. Taken together, our data in Fig. 2 established a strong correlation between antibody-induced downregulation of surface and total CTLA-4 and their irAE.

### pH-insensitive target binding of irAE-prone anti-CTLA-4 mAbs triggers lysosomal degradation of CTLA-4

**Fig. 3:**
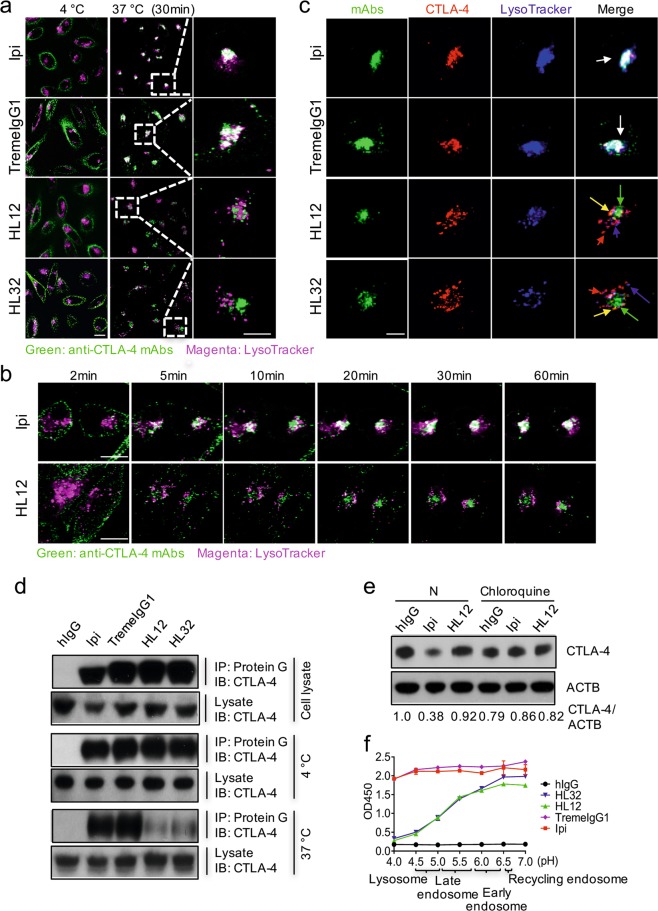
pH-insensitive target binding of irAE-prone anti-CTLA-4 mAbs triggers lysosomal degradation of CTLA-4. **a** Ipilimumab, TremeIgG1, HL12 or HL32 were labeled with AF488 and treated with CHO stable cell lines expressing hCTLA-4 at 4 °C. After extra antibodies were washed away, cells were incubated at 37 °C for 30 min and further stained with lysotracker. Co-localization between AF488-labeled anti-CTLA-4 mAbs and lysosomes was shown by confocal images (green: anti-CTLA-4 mAbs; meganta: Lysosomes; white: overlap of green and magenta). Scale bar: 10 µm. **b** Time-span of cells treated with Ipilimumab-AF488 and HL12-AF488 in (a) has been shown by representative confocal images. Scale bar: 10 µm. **c** Co-localization of AF488-labeled anti-CTLA-4 mAbs, lysosomes and orange-fluorescence protein (OFP)-tagged CTLA-4 in (**a**) was shown by representative confocal images (Green: anti-CTLA-4 mAbs; red: CTLA-4; blue: Lysosomes; white: overlapping of the three markers). Scale bar: 10 µm. **d** Stable HEK293T cell lines expressing hCTLA-4 were lysed and treated with anti-CTLA-4 mAbs for 1 h at 4 °C (Top panel). Cell surface CTLA-4 of HEK293T stable cell lines expressing hCTLA-4 was also labeled with anti-CTLA-4 mAbs at 4 °C for 30 min (middle panel). After washing out the unbound antibodies, cells were transferred to 37 °C for 1 h (bottom panel). Antibody-bound CTLA-4 was immunoprecipitated (IP) by protein G beads and tested by immunoblot (IB) with polyclonal anti-CTLA-4 antibodies. Input was 5% of total cell lysates. **e** With or without pre-treatment of lysosome inhibitor chloroquine, HEK293T cells transfected with hCTLA-4 were incubated with either control IgGFc, Ipilimumab or HL12 for 4 h. The CTLA-4 was analyzed by Immunoblot. **f** His-hCTLA-4 (0.5 µg/mL) was coated on ELISA plates and then different types of anti-CTLA-4 mAbs were added at 10 µg/mL in 1% BSA PBS with pH 4.0–7.0. Antibodies binding with CTLA-4 were measured then. Data are means of duplicate optical density at 450 nm. All the data in this figure have been repeated at least three times

As a pilot study to determine the mechanism of CTLA-4 degradation triggered by Ipilimumab, we treated 293T-CTLA-4 cell lines with Ipilimumab in the presence of either proteasome inhibitor MG132 or an inhibitor for lysosomal degradation (Chloroquine, CQ). As shown in Supplementary Information, Fig. S3a, downregulation of CTLA-4 by Ipilimumab was rescued by lysosome CQ but not MG132. These data raised the intriguing possibility that antibody-induced downregulation of surface CTLA-4 was due to lysosomal degradation of internalized CTLA-4. To test this hypothesis, we labeled anti-CTLA-4 antibodies with AF488 and incubated them with CTLA-4-expressing CHO cells at 4 °C first and then washed away all unbound antibodies. As shown in Fig. 3a left panel, all antibodies uniformly labeled cell surface CTLA-4 (green circles). Then we switched the temperature to 37 °C for 30 min in order to promote internalization, and observed the fate of antibody bound to cell surface CTLA-4. Lysosomes were labeled with lysotracker. As expected, all anti-CTLA-4 antibodies are internalized after CHO cells were switched to 37 °C. However, the internalized antibodies have different destination inside the cells. Cell surface-bound Ipilimumab and TremeIgG1 colocalized with lysotracker (Fig. 3a, middle and right panel). Time-span images showed that Ipilimumab started to merge within lysosome between 5-10 min after incubation at 37 °C and this transport largely reached a plateau within 30 min, and essentially, all the antibodies stayed within the lysosomes throughout the 60 min period of observation time (Fig. 3b, upper panels). In contrast, while HL12 was internalized within 2 min, they largely stayed away from lysosome throughout the observation period (60 min for HL12 and 30 min HL32) (Fig. 3a, b).

To determine whether the trafficking of anti-CTLA-4 antibodies reflects that of CTLA-4, we took advantage of the fact that CTLA-4 expressed on CHO cells has an orange fluorescent protein (OFP) tag which allows us to simultaneously follow the destination of antibodies and CTLA-4 in the CHO cells. Again, lysotracker was used to localize the lysosomes. As shown in Fig. 3c, essentially all Ipilimumab and TremeIgG1 were colocalized with CTLA-4 and lysotracker (white arrows in Fig. 3c). These data suggest that these two irAE-prone antibodies continuously associated with and drove CTLA-4 molecules into the lysosomes. Surprisingly, after CHO-CTLA-4 cells that had been incubated with HL12 and HL32 at 4 °C were shifted to 37 °C, the overwhelming majority of antibody-containing vesicles are devoid of CTLA-4, and few, if any antibody was found in the lysosome. These data suggest that while irAE-prone antibodies remain bound with intracellular CTLA-4 and drive CTLA-4 into lysosomes, non-irAE-prone antibodies may dissociate from CTLA-4 after endocytosis to avoid lysosomal targeting. To substantiate this possibility, we incubated antibodies with 293T-CTLA-4 at 4 °C, for 30 min. After washing away unbound antibodies, the cells were incubated at 4 °C or 37 °C for 1 h before they were lysed for immunoprecipitation with protein G beads to capture pre-existing antigen–antibody complex. As shown in Fig. 3d, at 4 °C, all antibody remained bound to CTLA-4 on CHO cells. However, after incubation at 37 °C, irAE-prone mAbs Ipilimumab and TremeIgG1 pulled down large amounts of CTLA-4, while HL12 and HL32 showed minimal association with their target. These data, along with the confocal images in Fig. 3a–c, demonstrate that after endocytosis, HL12 and HL32 largely disassociated from CTLA-4, potentially allowing both antibodies and their target to escape lysosomal degradation. In contrast, Ipilimumab and TremeIgG1 remained bound to CTLA-4 and potentially directed it to lysosomal degradation. To test this hypothesis, we evaluated the impact of chloroquine, which inhibits lysosomal protein degradation. As shown in Fig. 3e, chloroquine selectively rescued CTLA-4 from degradation induced by Ipilimumab but has no effect on the CTLA-4 levels in either untreated 293T-CTLA-4 cells or those that were treated with HL12.

Antibody binding to cell surface molecules often triggers endocytosis of antigen–antibody complex, which were normally directed to degradation as the endosome matures into lysosomes. This process is associated with progressive acidification. At the same time, cell surface CTLA-4 spontaneously undergoes endocytosis and recycling.^35^ We therefore hypothesize that HL12 and HL32 disassociated from CTLA-4 because they bound to CTLA-4 in a pH-dependent manner. To test this hypothesis, we tested the binding of CTLA-4 protein to anti-CTLA-4 antibodies at different pH. As shown in Fig. 3f, while binding of Ipilimumab and TremeIgG1 to CTLA-4 was largely intact under various pH ranging from pH 4 to pH 7, binding of HL12 and HL32 to CTLA-4 was progressively reduced once pH drops below 6.5, which is the pH of early endosomes. Extensive antibody titration further substantiated the observation (Supplementary Information, Fig. S3b). Thus, over a 10,000-fold range, Ipilimumab and TremeIgG1 exhibited essentially the same binding at pH 7 and pH 5.5. At lysosomal pH of 4.5, a slight reduction of less than 10-fold was observed when compared to pH 7. In contrast, HL12 and HL32 were highly pH-sensitive with 10–100-fold reduction at pH 5.5 and 100–1000-fold reduction at pH 4.5.

**Fig. 4:**
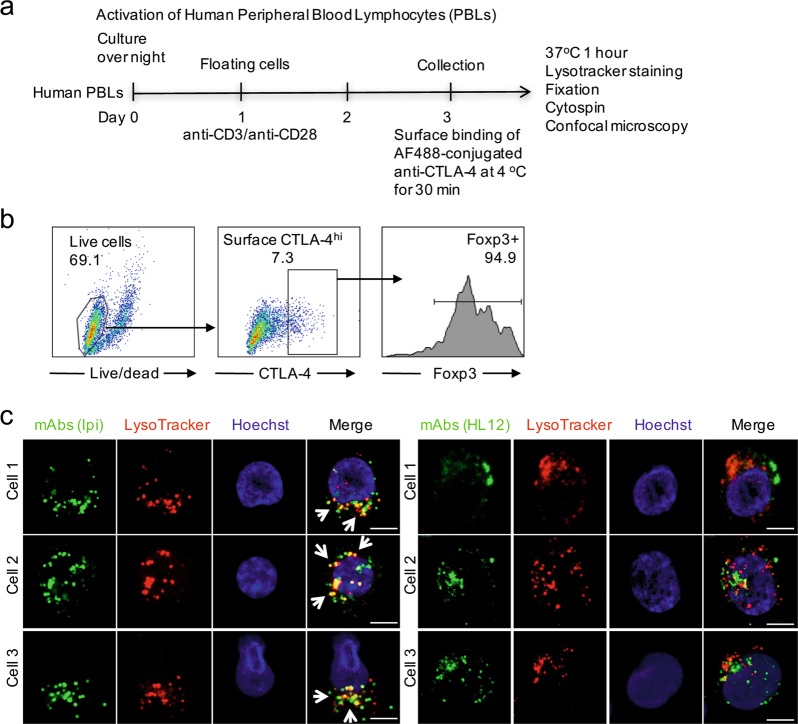
Distinct intracellular tracking of Ipilimumab and HL12 in human Treg. **a** Diagram of experimental design. **b** Activated PBLs were stained with surface markers (CD45, CD4 and CD8) and anti-CTLA-4 antibody BNI3. Cells were then fixed/permeabilized for anti-FOXP3 antibody staining. CTLA-4^hi^ CD4 cells were gated and analyzed for expression of FOXP3. **c** Anti-CD3/CD28-activated human PBLs were kept in 4 °C for half an hour. After that, cells were incubated with AF488-conjugated Ipilimumab or HL12 first at 4 °C (1 h) and then switched to 37 °C for 1 h after washing away unbound antibodies. Cells were stained with Lysotracker-Red DND-99 for 5 min, and fixed with 4% PFA for 5 min at RT. Fixed cells were quickly cytospun for confocal imaging. Data shown are three representative CTLA-4^hi^ Tregs in each treatment and have been reproduced in two independent experiments. Similar data were obtained when activated PBLs were attached to glass slides via poly-lysine and incubated with anti-CTLA-4 antibodies. Scale bar: 5 µm

To confirm the distinct trafficking of pH-sensitive and insensitive antibodies in human Treg, we activated human T cells with anti-CD3 and anti-CD28 and analyzed trafficking of Ipilimumab and HL12 in CTLA-4^hi^ cells as diagramed in Fig. 4a. Since 95% of CTLA-4^hi^ cells expressed FOXP3 (Fig. 4b), they were considered as Tregs. Therefore, we compared trafficking of Ipilimumab and HL12 in human Treg by confocal microscopy. As shown in Fig. 4c, within 1 h of incubation at 37 °C, the majority of Ipilimumab have moved to lysosomes. In contrast, most HL12-containing vesicles were largely negative for lysotrackers.

We further evaluated if the preformed complex would dissociate in the acidic environment by allowing antibodies to bind to CTLA-4 at neutral pH and then wash the preformed complex with acidic buffers. As shown in Supplementary Information, Fig. S3c, HL12 and HL32 dissociated from CTLA-4 at pH < 6.0. In contrast, no disassociation was observed between Ipilimumab and TremeIgG1 with CTLA-4- from pH 4 to pH 7. Taken together, our data suggest differential pH sensitivity as a potential mechanism by which irAE-prone and non-irAE-prone anti-CTLA-4 antibodies differ.

### LRBA-dependent recycling prevents antibody-induced CTLA-4 degradation

**Fig. 5:**
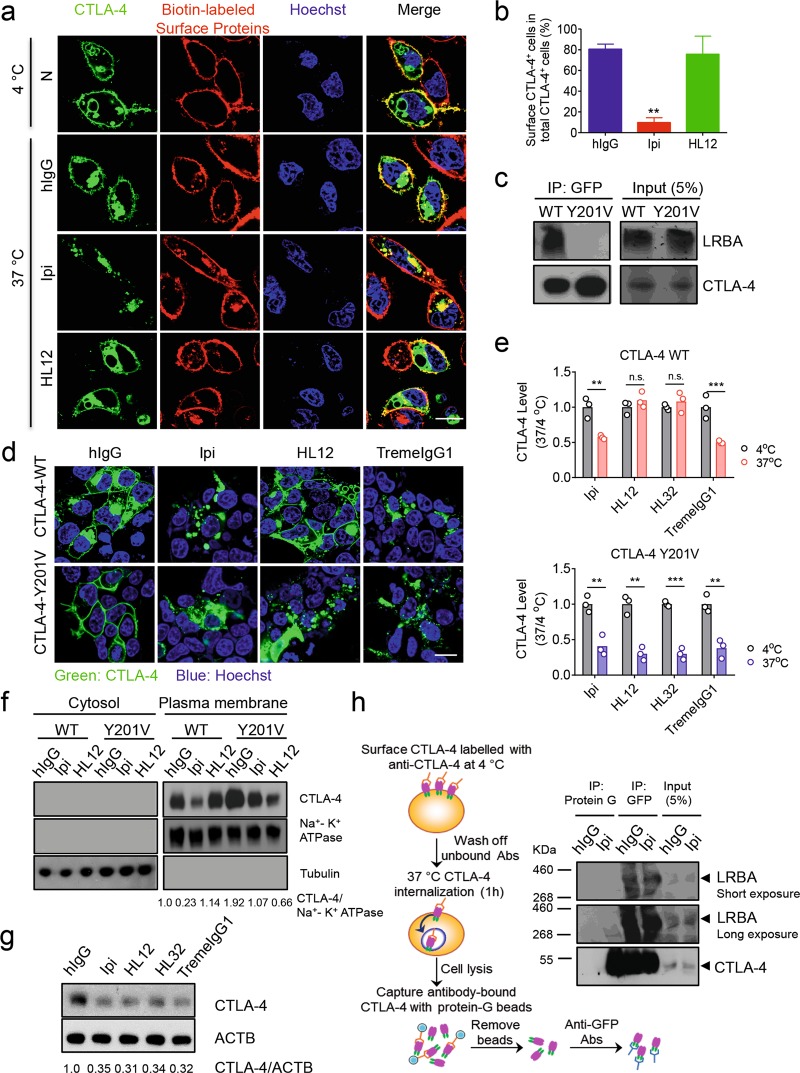
LRBA-dependent recycling prevents antibody-induced CTLA-4 degradation. **a** CHO cells were transiently transfected with human GFP-tagged CTLA-4-expressing constructs for 24 h. Cell surface proteins were biotinylated and stained with Avidin-AF594 at 4 °C. This converted surface CTLA-4 into yellow color prior to antibody-treatment. After that, cells were incubated at 37 °C for 2 h with anti-CTLA-4 antibodies prior to confocal imaging. Nuclei were labeled with Hoechst. Note the predominantly intracellular location of cell surface CTLA-4 after Ipilimumab but not HL12 treatment. Scale bar: 10 µm. **b** The ratio of surface CTLA-4-positive cells versus total CTLA-4-positive cells was quantified by counting 30 CTLA-4-positive cells per group. **c** HEK293T cells were transfected with constructs of GFP-fused WT-CTLA-4 or CTLA-4 mutant (Y201V). Anti-GFP immunoprecipitates (IP) were immunoblotted with antibodies against LRBA and CTLA-4. Input was 5% of total cell lysates. **d** HEK293T cells transfected with GFP-fused WT or Y201V mutant CTLA-4 were incubated with control hIgGFc, Ipilimumab, HL12 or TremeIgG1 for 4 h. Representative confocal images of CTLA-4 have been shown. Scale bar: 10 µm. **e** HEK293T cells transfected with GFP-fused WT hCTLA-4 or GFP-fused Y201V hCTLA-4 were treated with Ipilimumab, TremeIgG1, HL12 or HL32 at 4 °C. After 30 min, while half amount of the cells was kept at 4 °C, the other half amount of the cells was moved to the 37 °C for another 2 h. After washing out unbound antibodies, cell surface CTLA-4 was detected by an AF488-conjugated anti-human IgGFc antibody at 4 °C and analyzed by flow cytometry. Data shown are summary of relative mean fluorescence intensities of cell surface CTLA-4 after normalization against the AF488 fluorescence measured at 4 °C, the mean of which are artificially defined as 1.0. **f** HEK293T cells transfected with GFP-fused WT or Y201V mutant hCTLA-4 were incubated with control hIgG, Ipilimumab or HL12 for 4 h. Plasma membrane proteins were isolated and the cell surface CTLA-4 was detected by Immunoblot. **g** Transfected CTLA-4-Y201V mutant cells in (**f**) were incubated with control hIgGFc, Ipilimumab, TremeIgG1, HL12 or HL32 respectively for 4 h. The CTLA-4 protein level was analyzed by western blot. Data in (**b**) are mean ± SEM, while those in e are plot with individual values. *p < 0.05, **p < 0.01, ***p < 0.001, ****p < 0.0001. **h** Ipilimumab disrupts association of endocytosed CTLA-4 with LRBA. As diagrammed on the left, CHO cells stably expressing GFP-tagged CTLA-4 were incubated with Ipilimumab at 4 °C to capture cell surface CTLA-4. After removing unbound antibodies, the cells were shifted to 37 °C for 1 h to allow endocytosis. The cells were then lysed and the lysates were incubated with protein G beads to capture Ipilimumab-bound CTLA-4 and associated molecules. Once the Ipilimumab-bound molecules were removed, the supernatants were incubated with anti-GFP antibodies, followed by protein G beads to capture CTLA-4 that were not bound to Ipilimumab. The immunoprecipitates and input cell lysates were probed with either anti-CTLA-4 or anti-LRBA (right). Images in (**a**) are representative of those from two independent experiments. Analysis data in (**b**) are means of two independent experiments involving 4 independent cultures per group. Representative images in (**c**) and (**d**) are from three independent experiments. Data in (**e**) are two independent experiments, two samples per group. Representative data of three independent experiments in (**f**–**h**) were shown

In order to directly monitor the fate of cell surface CTLA-4, we transfected CHO cells with EGFP-tagged CTLA-4 and then biotinylated the cell surface proteins. After staining with AF549-labeled streptavidin, the cell surface CTLA-4 exhibited a colocalized signal. As expected, when biotinylated cells were maintained at 4 °C, all colocalized signals are on the cell surface. We then shifted the cells to 37 °C and incubated the GFP-CTLA-4-transfected 293T cells with control IgG, Ipilimumab, TremelgG1, or HL12 and observed the cell surface CTLA-4 by confocal microscope. In the control hIgG-treated cells, most cell surface CTLA-4 remained on the cell surface, although a fraction of cell surface CTLA-4 was internalized, which is expected based on constitutive internalization of CTLA-4. While essentially all cell surface CTLA-4 has become intracellular after Ipilimumab treatment, HL12-treated cells had high levels of cell surface CTLA-4 (Fig. 5a, b). Considering the fact that HL12 had undergone rapid internalization (Fig. 3a, b), it is intriguing that the cell surface CTLA-4 in HL12-treated cells may be derived from recycling internalized CTLA-4 molecules. To confirm this possibility, we took the advantage of the CTLA-4 mutant (Y201V) which cannot be recycled due to lack of interaction with LRBA, an essential mediator for CTLA-4 recycling.^36^ We first confirmed the effect of the Y201V mutation on LRBA association by co-IP (Fig. 5c). To confirm the critical role for CTLA-4 recycling on its resistance to HL12-induced downregulation, we transfected 293T cells with GFP-tagged either WT or Y201V mutant CTLA-4 and compared their response to anti-CTLA-4 mAbs. Both WT and mutant CTLA-4 were tagged with GFP for tracking their cellular distribution. As shown in Fig. 5d, e, irAE-prone Ipilimumab- or TremeIgG1-treated WT-CTLA-4 transfectants are largely devoid of cell surface CTLA-4, while non-irAE-prone HL12- or HL32-treated WT-CTLA-4-transfected cells retained cell surface CTLA-4. In contrast, all anti-CTLA-4 mAbs abolished cell surface CTLA-4 in Y201V-transfectants. To substantiate the differential effects of the anti-CTLA-4 mAbs on cell surface CTLA-4, we measured membrane-associated CTLA-4 in WT and Y201V mutant-transfected cells by immunoblot. As expected, WT CTLA-4 was present in the plasma membrane and was downregulated after Ipilimumab treatment but not after HL12 treatment. Remarkably, with mutation Y201V, HL12 is even more potent than Ipilimumab in reduction of plasma membrane-associated CTLA-4. The reduction of cell surface CTLA-4 (Fig. 5d–f) was accompanied by a reduction of total CTLA-4Y201V levels, as visualized in Fig. 5g. These data suggest that non-irAE-prone anti-CTLA-4 mAbs were prevented from downregulating CTLA-4 by LRBA-dependent recycling, and this mechanism was rendered inoperative by Ipilimumab and TremeIgG1.

A potential mechanism by which Ipilimumab prevents LRBA-dependent recycling is to prevent CTLA-4 from binding to LRBA. Since Ipilimumab remains bound to CTLA-4 after endocytosis, we compared Ipilimumab-bound CTLA-4 with those that had not interacted with Ipilimumab for their association with LRBA in 293T cells expressing GFP-tagged CTLA-4, using a strategy diagrammed in Fig. 5h, left panel. Thus, the Ipilimumab-bound CTLA-4 molecules were first pulled down by protein G, while those that had not interacted with Ipilimumab were sequentially pulled down using the anti-GFP antibodies. The amount of LRBA in the immunoprecipitates was determined by immunoblot. As shown in Fig. 5h right panel, the Ipilimumab-bound CTLA-4 did not associate with LRBA, while those that had not interacted with Ipilimumab associated with CTLA-4. Therefore, Ipilimumab binding prevented CTLA-4:LRBA association.

### CTLA-4 recycling prevents irAE of anti-CTLA-4 antibodies

**Fig. 6:**
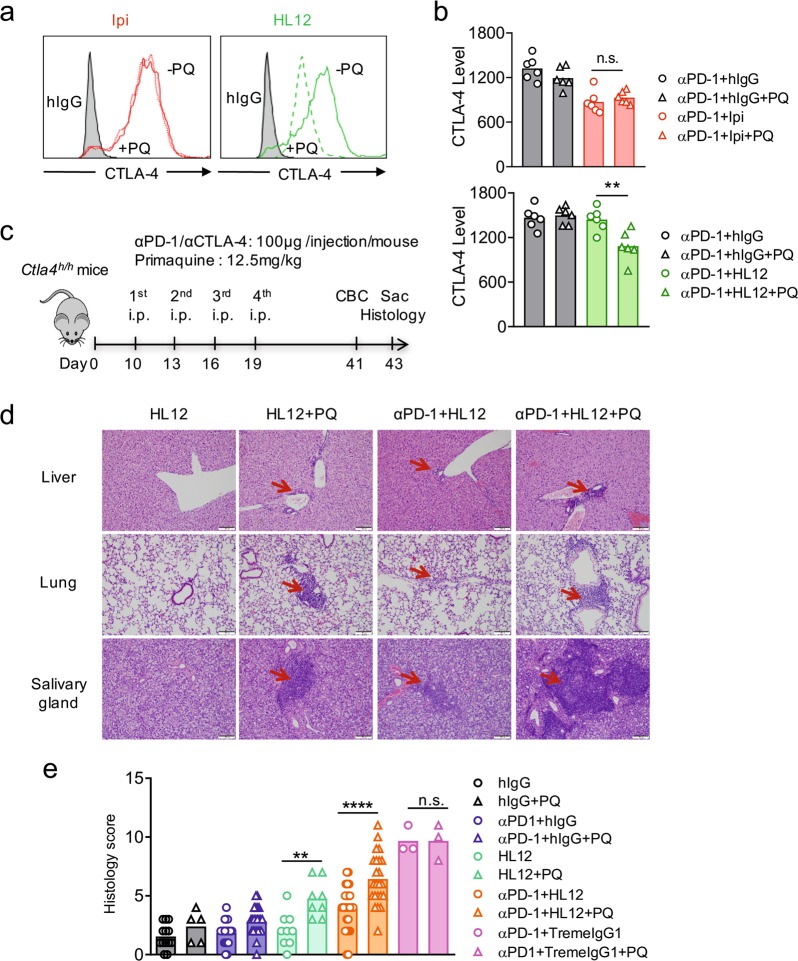
Disruption of CTLA-4 recycling underlies irAE of anti-CTLA-4 antibodies. **a** Stable CHO cell lines expressing hCTLA-4 were incubated with Ipilimumab or HL12 or control IgGFc at 37 °C ± primaquine (PQ) for 2 h. After washing out unbound antibodies, cell surface CTLA-4 was detected with an AF488-conjugated anti-human IgGFc antibody and analyzed by flow cytometry. Shaded grey histograms show background staining of cells incubated with hIgGFc control, while colored histograms depict cell surface CTLA-4 when cells were treated with anti-CTLA-4 in the presence (broken lines) or absence of PQ (solid lines). **b** Ten-day old *Ctla4*^*h/h*^ mice (body weight: 4.5–5.3 g; *n* *=* 6) were treated with anti-PD-1 (100 μg/mouse) in the presence or absence of PQ (12.5 mg/kg, i.p.). 24 h later, mice received i.p. injection of 100 μg control IgGFc, Ipilimumab or HL12 ± PQ (12.5 mg/kg). After 4 h, mice were sacrificed for flow cytometry of cell surface and total CTLA-4 on/in Tregs isolated from lung. Similar data were obtained from spleen Treg (not shown). **c** Diagram of PQ treatment in the irAE model. Ten-day old *Ctla4*^*h/h*^ mice (body weight: 4.5–5.3 g; *n* *=* 5–25 mice per group) were treated with control hIgG, hIgG plus anti-PD-1, HL12, HL12 plus anti-PD-1 or TremeIgG1 plus anti-PD-1, respectively, at a dose of 100 μg/mouse/injection on days 10, 13, 16 and 19 after birth, with or without a combination PQ at 12.5 mg/kg. Mice were sacrificed on day 43 for the histopathology study. **d** Representative images of H&E stained paraffin sections from the liver, lung and salivary gland on (**c**) are shown. Representative inflammatory foci are indicated with arrows. Scale bar: 200 μm. **e** Composite histopathology scores of the organs and glands in (**d**). Three mice in the TremeIgG1-treated groups died before reaching the endpoint and thus did not contribute to their histopathology scores. Data were analyzed by one-way ANOVA with Bonferroni’s multiple comparisons. Data shown are means, with each point representing score of individual mice in the group. *p < 0.05, **p *<* 0.01, ***p < 0.001, ****p *<* 0.0001. Representative data of two independent experiments in (**a**) and (**b**) were shown. The samples in (**d**) and (**e**) were collected from three independent experiments and have been scored double blind

Primaquine (PQ) is an inhibitor of endocytic recycling.^38^ Therefore, we used it as a probe for contribution of recycling on cell surface CTLA-4 levels. As shown in Fig. 6a, incubation with PQ induced rapid loss of surface CTLA-4 in HL12-treated CTLA-4-expressing CHO cells, suggesting that a large proportion of surface CTLA-4 was continuously internalized and recycled in HL12-treated cells. In contrast, PQ had no effect on surface CTLA-4 in Ipilimumab-treated CHO cells, which suggest that very little recycling occurs in this setting (Fig. 6a). To test this notion in vivo, we injected PQ in conjunction with anti-PD-1 and anti-CTLA-4 and measured CTLA-4 levels 4 h after treatment. As shown in Fig. 6b, Ipilimumab downregulated CTLA-4 in Treg from lung, and this downregulation was largely unaffected by PQ. As expected, HL12 had no effect on CTLA-4 levels. However, the addition of PQ caused significant downregulation.

The selective reduction of CTLA-4 by PQ in HL12-treated mice provided us with an opportunity to test if continuous recycling of CTLA-4 explained lack of irAE by HL12. We treated mice with PQ in conjunction with anti-PD-1 and three different anti-CTLA-4 antibodies, as depicted in Fig. 6c. An example for the PQ effect on HL12 treatment is presented in Fig. 6d, and a summary of the inflammation scores in these organs for three different anti-CTLA-4 is presented in Fig. 6e. We found a dose that does not cause toxicity in mice (our unpublished observation), PQ increased the toxicity of HL12, either as monotherapy or as combination therapy in conjunction with anti-PD-1. This is highly selective as PQ did not exacerbate irAE-caused by combination of anti-PD-1 plus TremeIgG1 (Fig. 6e).

### Increasing pH sensitivity of an anti-CTLA-4 antibody attenuates its irAE

Introduction of histidine to the complementarity-determining regions (CDRs) has been shown to increase pH sensitivity of the antibody–antigen interaction.^39^ To determine whether pH sensitivity of anti-CTLA-4 mAbs contributes to irAEs, we replaced tyrosine with histidine in the CDRs of TremeIgG1 and generated 6 variants of TremeIgG1, which we called Ab154-159 (Supplementary Information, Table S1). Affinities of the antibodies to CTLA-4 were measured using surface plasma resonance (SPR) and the data are summarized in Supplementary Information, Table S1. These data demonstrated that all but one antibody (Ab155) exhibited high affinity binding to CTLA-4, although the variants did exhibit somewhat reduced affinity to CTLA-4 when compared with WT TremeIgG1.

**Fig. 7:**
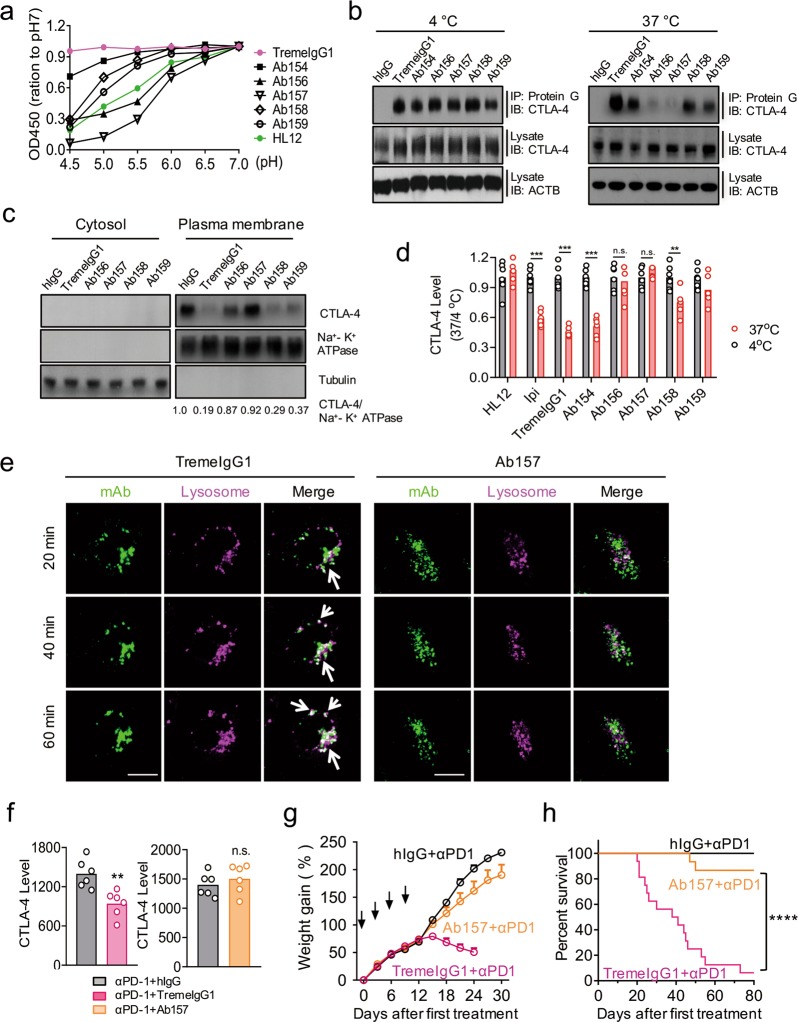
Engineering antibody variants with increased pH sensitivity to attenuates irAEs. **a** His-hCTLA-4 (0.5 μg/mL) was pre-coated on ELISA plates. HL12, TremeIgG1 and its variants (Ab154-Ab159) were added at 1 μg/mL in 1% BSA PBS with pH ranging from pH 4.5 to 7.0. Antibodies bound to CTLA-4 were measured using horse-radish peroxidase-labeled anti-human IgG antibodies. Data shown are the means of duplicate optical density at 450 nm and shown as the normalization with the OD450 value at pH 7.0. **b** Stable HEK293T cell lines expressing hCTLA-4 was incubated with hIgG, TremeIgG1 and its variants at 4 °C for 30 min. After washing out the unbound antibodies, cells were transferred to 37 °C for 1 h. Antibody-bound surface CTLA-4 was immunoprecipitated (IP) by protein G beads and tested by immunoblot (IB). **c** Stable HEK293T cell lines expressing hCTLA-4 were incubated with either control IgG, TremeIgG1 or its variants for 4 h. Plasma membrane and cytosolic fractions were isolated for detection of CTLA-4 by immunoblot. **d** Human CTLA-4-expressing CHO cells were treated with HL12, Ipilimumab, TremeIgG1 and its variants at 4 °C. After 30 min, half of the cells were kept at 4 °C, and the other half were switched to the 37 °C for another 2 h. Cell surface CTLA-4 was detected by an AF488-conjugated anti-human Fc antibody at 4 °C and analyzed by flow cytometry. Mean fluorescence intensity of AF488 fluorescence at 4 °C is artificially defined as 1. **e** Stable CHO cell lines expressing hCTLA-4 was incubated with either TremeIgG1-AF488 or variant Ab157-AF488 at 4 °C for 30 min. After unbound antibodies were washed away, the cells were transferred to 37 °C for 20, 40 and 60 min. Cells were further stained with lysotracker to visualize the co-localization between anti-CTLA-4 and lysosomes. Data shown are representative confocal images (Green: anti-CTLA-4 mAbs; megenta: Lysosomes; white: overlapping of the two colors). Scale bar: 10 µm. **f** Ten days of *Ctla4*^*h/h*^ mice (body weight: 4.5–5.3 g; *n* = 6) were treated with anti-PD-1 at 100 μg/mouse. 24 h later, the mice received either control IgG, TremeIgG1 or variant Ab157 (100 μg/mouse). 4 h after injection of anti-CTLA-4 antibodies, cell surface CTLA-4 on Tregs isolated from mice lung were evaluated by flow cytometry. **g**, **h** Ten-days old C57BL/6 *Ctla4*^*h/h*^ mice (body weight: 4.5–5.3 g) were treated with control hIgGFc plus anti-PD-1, TremeIgG1 plus anti-PD-1, or Ab157 plus anti-PD-1, at a dose of 100 μg/mouse/injection on days 10, 13, 16 and 19 of birth. Mice were observed for body weight gain (**g**) and morbidity and mortality (**h**). Data shown in (**g**) are means ± S.E.M. of % weight gain following the first injection, *n* = 15–16. Statistical significance in the difference between TremeIgG1 and Ab157 was determined by two-way repeat measurement ANOVA. (**h**) Kaplan–Meier survival analyses. Statistical significance in the difference between TremeIgG1 and Ab157 was determined by log-rank test. Data are mean ± SEM. *p < 0.05, **p < 0.01, ***p < 0.001, ****p < 0.0001. Data in (**a**) have been reproduced in four independent experiments. Representative data of three independent experiments in (**b**) and (**c**) are presented. Data in (**d**) are combined from three independent experiments, each includes two samples per group. Representative data of two independent experiments in (**e**) and (**f**) were shown. Data in (**g**) and (**h**) are combined from two independent experiments

We then measured the binding of TremeIgG1 and its variants to CTLA-4 at various pH by ELISA. HL12 was included as a pH-dependent antibody control. As shown in Fig. 7a, WT TremeIgG1 showed similar binding to CTLA-4 at all pH tested, while all variants exhibited pH-sensitive binding of different degrees. Among them, Ab156 and Ab157 were more sensitive than HL12 to low pH, while Ab154, Ab158, and Ab159 were less so. At pH value equal to that in late endosome (pH 5.5), HL12, Ab156 and Ab157 showed 50% or less binding to CTLA-4 when compared to that at pH 7.0, while Ab158 and Ab159 still exhibited 75% or more binding (Fig. 7a; Supplementary Information, Fig. S4a). Similar pH-dependent binding was observed when pH was changed after binding was established at pH 7.0 (Supplementary Information, Fig. S4b). Moreover, the pH-dependence is not due to the low affinity of antibody at neutral pH, as when compared to Ab154, antibodies Ab158 and Ab159 showed substantial higher pH sensitivity despite higher affinity (Fig. 7a; Supplementary Information, Table S1).

To test if pH sensitivity causes the antibodies to dissociate from CTLA-4 after endocytosis, we compared their binding to CTLA-4 after they were incubated at either 4 °C or 37 °C. At 4 °C, all antibodies bound comparably to CTLA-4, as revealed by immunoprecipitation followed by immunoblot (Fig. 7b, left panel). After incubating at 37 °C for 1 h, the amount of antibody-associated CTLA-4 correlates with pH sensitivity of the antibodies. Thus, while high amounts of CTLA-4 remained associated with TremeIgG1, measurably reduced amounts were found to bind to Ab154, Ab158 and Ab159, the antibodies that showed partial pH sensitivity. Much less CTLA-4 was associated with Ab156 and Ab157, the two most pH-sensitive antibodies, when compared with WT or other less pH-sensitive variants (Fig. 7b). We evaluated plasma membrane-associated CTLA-4 levels following treatment of TremeIgG1 or its variants by immunoblot of cellular fractions. This assay revealed significant differences in the remaining plasma membrane-associated CTLA-4 after treatment with different variants, with plasma membrane-associated CTLA-4 being inversely proportional to pH sensitivity (Fig. 7c). Flow cytometry data showed that cell surface level of CTLA-4 was unaffected by Ab156 and Ab157 (Fig. 7d). A modest reduction of surface CTLA-4 was induced by Ab158 and 159. Ab154 was nearly as potent as TremeIgG1 in reducing cell surface CTLA-4 (Fig. 7d). Thus, the reduction of cell surface CTLA-4 by the anti-CTLA-4 antibody variants correlates negatively with the pH-dependence in their bindings with CTLA-4 molecules.

We also evaluated internalization of AF488-labeled TremeIgG1 and Ab157 kinetically using confocal microscopy. As shown in Fig. 7e, unlike TremeIgG1, Ab157 did not translocate to lysosomes and thus should be unable to direct CTLA-4 for lysosomal degradation, as demonstrated by its inability to downregulate CTLA-4 on 293T cells. Furthermore, we evaluated the effect of TremeIgG1 and Ab157 on CTLA-4 levels of Tregs isolated from lung of *Ctla4*^*h/h*^ mice. While TremeIgG1 significantly reduced surface CTLA-4 in Tregs, Ab157 had no effects (Fig. 7f).

To test if pH sensitivity confers better safety, we compared TremeIgG1 and its pH-sensitive variant, Ab157, in young *Ctla4*^*h/h*^ mice that also received anti-PD-1 treatment to sensitize them to irAE, as depicted in Fig. 6c. We followed mouse weight gain and survival after 4 treatments. As shown in Fig. 7g, h, anti-PD-1+TremeIgG1 induced rapid weight loss in young mice, which was followed by progressive mortality over a two-month period, resulting in approximately 90% mortality rate. In contrast, all but 2/15 mice that received anti-PD-1+Ab157 survived the entire observation period (Fig. 7h), with only modest reduction in growth rate. These data demonstrate that increasing pH sensitivity can confer dramatic improvement of the safety profiles of anti-CTLA-4 antibodies.

### pH sensitivity confers improved therapeutic efficacy of anti-CTLA-4 antibodies

**Fig. 8:**
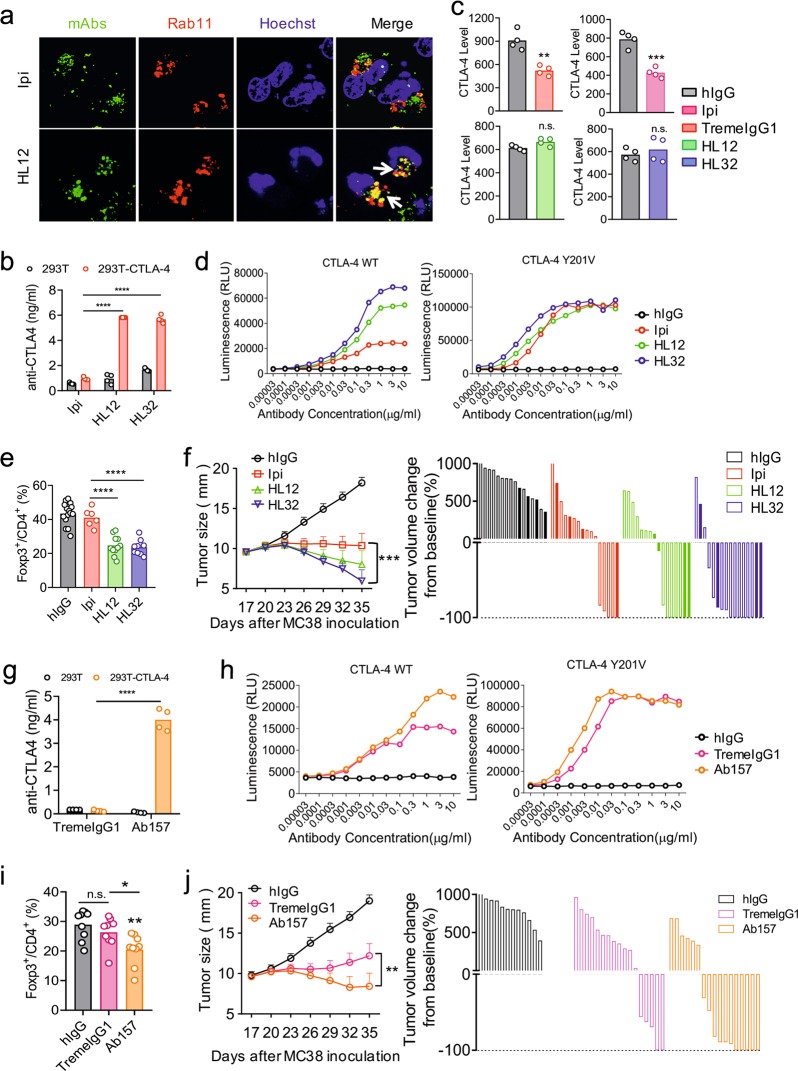
pH sensitivity confers improved therapeutic effect of anti-CTLA-4 antibodies. **a** CHO cells were transfected with human CTLA-4 constructs and ds-Red-tagged human Rab11 constructs. After 24 h, cells were incubated with either Ipilimumab-AF488 or HL12-AF488 at 4 °C for 30 min. After extra antibodies were washed away, cells were incubated at 37 °C for 1 h. Representative confocal images of co-localization between AF488-labeled antibodies and Rab11 are shown (green: anti-CTLA-4 mAbs; red: Rab11; blue: nuclei; yellow: overlapping between anti-CTLA-4 mAbs and Rab11). Scale bar: 10 µm. **b** HEK293T or HEK293T stable cell lines expressing hCTLA-4 were treated with Ipilimumab, HL12 or HL32 at 4 °C for 30 min. After washing out unbound antibodies, the cells were incubated at 37 °C for 4 h. Culture medium of treated cells was collected and the concentrations of released anti-CTLA-4 antibodies in the medium were measured by ELISA. **c** MC38 tumor grown in *Ctla4*^*h/h*^ mice were digested with collagenase IV, hyaluronidase and deoxyribonuclease to prepare single cell suspensions. The cells were treated with either control hIgGFc or anti-CTLA-4 mAbs for 4 h in vitro. Surface CTLA-4 of tumor-infiltrating Tregs was measured by flow cytometry in the presence of excess antibodies to avoid the influence of antibody masking. **d** ADCC activities of Ipilimumab, HL12 or HL32 were tested using an in vitro ADCC bioassay. Data shown are luminescence units emitted from reporter cells expressing FcγRIIIA. HEK293T stable cell lines expressing hCTLA-4 WT or hCTLA-4-Y201V were used as target cells. **e** MC38-bearing *Ctla4*^*h/h*^ mice (*n* *=* 6–15) were treated with either control hIgG, Ipilimumab, HL12 or HL32 (100 μg/mouse) on day 14 after tumor inoculation. Selective depletion of Treg cells in the tumor microenvironment was determined by the percentage of Treg cells among CD4 T cells at 16 h after antibody treatment. **f** MC38 tumor-bearing *Ctla4*^*h/h*^ mice (*n* = 16) were i.p. treated with either control hIgG, Ipilimumab, HL12 or HL32 (30 μg/mouse). 12/16 mice in each group received antibodies on day 17 and day 20 after tumor inoculation (shown as unfilled bars), while 4/16 mice in each group received 4 injections, respectively on days 17, 20, 23 and 26 after tumor inoculation (shown as solid bars). The data are from day 17 to day 35 when some mice in control group has reached tumor size endpoint. The line graphs in the left panel show mean tumor sizes, mean diameters ± S.E.M. The waterfall graphs on the right show either grow (above *X*-axis) or regression (underneath *X*-axis) of individual tumors. All data from three independent experiments are included. The tumor volume immediately prior to antibody treatment was defined as baseline. **g** HEK293T or HEK293T stable cell lines expressing hCTLA-4 cells were treated with TremeIgG1 or its pH-sensitive variant Ab157 at 4 °C for 30 min. After washing out unbound antibodies, the cells were incubated at 37 °C for 4 h. Then, the culture medium of treated cells was collected and the concentration of released anti-CTLA-4 antibodies in the medium were measured by ELISA. **h** ADCC activities of TremeIgG1 and variant Ab157 were tested using an in vitro ADCC bioassay. Data shown are luminescence units emitted from reporter cells expressing FcγRIIIA. HEK293T stable cell lines expressing hCTLA-4 WT or hCTLA-4-Y201V were used as target cells. **i** MC38-bearing *Ctla4*^*h/h*^ mice (*n* = 9) were treated with either TremeIgG1 or variant Ab157 (30 μg/mouse) on day 14 after tumor inoculation. Selective depletion of Treg cells in the tumor microenvironment was determined by the percentage of Treg cells among CD4 T cells at 24 h after antibody treatment. **j** MC38-bearing-Ctla4^*h/h*^ mice (*n* = 12–17) were treated with TremeIgG1 and variant Ab157 (30 μg/mouse) on day 17 and day 20 after tumor inoculation. Since experiments in (**j**) were performed concurrently with two experiments in (**f**), control hIgG group in (**j**) is a subset of (**f**). Representative images in (**a**) are from two independent experiments. Presented data in (**b**) and (**g**) are from two independent experiments each including two samples per group. Representative data of two independent experiments in (**c**), (**d**) and (**h**) were shown. Data in (**e**), (**i**) and (**j**) were combined with two independent experiments. Those in (**f**) were combined from three experiments. *p < 0.05, **p < 0.01, ***p < 0.001, ****p < 0.0001

Recent studies have demonstrated that anti-CTLA-4 mAbs confer their CITE by selective depletion of Treg within the tumor microenvironment (TME) through antibody-dependent cellular cytotoxicity/antibody-dependent cellular phagocytosis (ADCC/ADCP).^29–32,40–42^ It is therefore of interest to consider how pH sensitivity affects the ADCC activity of anti-CTLA-4 antibodies. To address this issue, we considered whether pH sensitivity affects the bioavailability of anti-CTLA-4 antibodies and target density on tumor-infiltrating Tregs. Since we have shown in Figs 2–4 that pH-sensitive antibodies separated from antigens and were not targeted to lysosomes, we investigated if these antibodies may enter into recycling vesicles, which can be marked by anti-Rab11 antibodies. As shown in Fig. 8a, AF488-labeled HL12 entered into Rab11^+^ recycling vesicles, while AF488-labeled Ipilimumab, which were destined to lysosomes (Fig. 3), did not enter. To test if the antibodies are recycled as functional antibodies, we incubated Ipilimumab, TremeIgG1, HL12 and HL32 with either control 293T or CTLA-4-transfected 293T. After washing away unbound antibodies, the antibody-coated cells were incubated at 37 °C for 4 h before the supernatants were collected to measure anti-CTLA-4 antibodies. As shown in Fig. 8b, the supernatants of CTLA-4-expressing cells contained 3–5-fold higher levels of HL12 and HL32 than those from control cells. However, supernatant from CTLA-4^+^ and CTLA-4^−^ 293T cells contained comparable and barely detectable levels of Ipilimumab. We further validated the differential effect of the antibodies on CTLA-4 levels in the ex vivo tumor-infiltrating T cells by incubating the antibodies with a single cell suspension from MC38 tumor explants. As shown in Fig. 8c, while Ipilimumab and TremeIgG1 downregulated CTLA-4 in Tregs, HL12 and HL32 had no effect.

The increased bioavailability and increased CTLA-4 target density suggested that HL12 and HL32 may have better ADCC/ADCP activity than Ipilimumab. To measure ADCC activity of these antibodies, we adopted the ADCC reporting system of Promega Inc. and used CTLA-4-transfected CHO cells as targets. As shown in Fig. 8d (left panel), HL12 and HL32 achieved much higher maximal ADCC signal than Ipilimumab. To determine whether increased ADCC activity relates to CTLA-4 recycling, we also tested ADCC activity using CHO cells expressing CTLA-4 Y201V mutants. As shown in the right panel of Fig. 8d, all antibodies achieved similar maximal ADCC activity if CTLA-4 mutant-transfected cells were used as target. Thus, the higher ADCC activity of HL12 and HL32 than Ipilimumab toward WT CTLA-4 expressing cells is likely attributable to their preservation of CTLA-4 recycling.

To test the function of the antibodies on Treg depletion in tumor, we injected the antibodies into mice which were challenged with MC38 tumors 14 days previously. 16 h later, the tumors were harvested and the percentage of Treg among CD4 T cells were assessed by flow cytometry. As shown in Fig. 8e, while HL12 and HL32 significantly reduced Treg within 24 h, Ipilimumab did not deplete Treg at this time point. At 4 days after antibody treatment, a trend of Treg depletion was observed by Ipilimumab treatment, although it is still less effective than HL12 and HL32 (Supplementary Information, Fig. S5).

We have previously demonstrated that for various small tumors, Ipilimumab, HL12 and HL32 are comparable in their efficacy in inducing tumor rejection.^29^ Given the better efficacy of Treg depletion by HL12 and HL32, and given the critical role of Treg in suppressing anti-tumor T cell responses, we re-evaluated the efficacy of different anti-CTLA-4 antibodies in a more challenging setting, i.e., in mice that bare large tumors and thus with a well-established microenvironment. We treated mice with 2-4 doses of 1.5 mg/kg/dose of Ipilimumab, HL12, HL32, or IgG control on day 17 and day 20 after tumor inoculation (tumors had an average size of 10 mm in diameter). As shown in Fig. 8f, at these limiting doses, HL32 was significantly more effective than Ipilimumab. Thus, as shown in the left panel, Ipilimumab caused stagnation but not reduction of average tumor sizes, while both HL12 and HL32 induced clear reduction. Understandably, when treating large tumor with a limiting dose of antibodies, the responses are heterogeneous among mice within the same group. A waterfall graph in the right panel of Fig. 8f revealed that tumors in mice receiving HL12 or HL32 showed slower growth, more robust regression and higher rate of complete response. In particular, the complete response rates among mice receiving either HL12 or HL32 were 2–3-fold that of those receiving Ipilimumab.

To substantiate the significance of pH sensitivity and CITE, we compared TremeIgG1 and its pH-sensitive variant Ab157 for bioavailability in vitro, ADCC activity, Treg depletion and tumor rejection. Our data demonstrated that the pH-sensitive variant accumulated at higher levels in the supernatants of CTLA-4^+^ cell culture (Fig. 8g). Correspondingly, the pH-sensitive variants showed higher ADCC activity at saturating doses (Fig. 8h, left panel). This can be attributable to CTLA-4 recycling in the target cells, as the variant was no more effective than TremeIgG1 if CTLA-4 Y201V variant was expressed on target cells (Fig. 8h, right panel). Correspondingly, the pH-sensitive variant was more effective in Treg depletion in vivo (Fig. 8i). More importantly, the pH-sensitive variant was significantly more effective than the TremeIgG1 in rejection of large established tumors (Fig. 8j). The percentage of mice receiving Ab157 with complete tumor rejection on day 41 is 2.5 fold of those which received TremeIgG1. Combining data in Fig. 8f, j revealed that at the limiting dose, the complete response rate among mice receiving pH-insensitive antibodies (Ipilimumab and TremeIgG1) was 15% (5/33), while the rate for those that received pH-sensitive antibodies (Ab157, HL12 and HL32) reached 39% (19/49). The difference is statistically significant (*p* = 0.008, Chi-Square test).

## Discussion

Anti-CTLA-4 antibody was the first of several T-cell-targeting antibodies that have been approved for clinical use for cancer immunotherapy. Nevertheless, CTLA-4-targeting reagents have not reached its full potential as evidenced by relatively low response rates, limited clinically approved indications, and high rates of irAEs. Here we describe a new paradigm for developing safer and more effective anti-CTLA-4 antibodies with broad implications for cancer immunotherapy.

### CTLA-4 downregulation and irAE

It is clear that, for the anti-CTLA-4 antibodies that have sufficient clinical data, including Ipilimumab and Tremelimumab, CTLA-4-targeting induced high rates of irAE. For instance, 75–90% of patients who received 10 mg/kg or more of Ipilimumab developed irAE of any grades.^43^ In the neo-adjuvant setting, combination of Ipilimumab and Nivolumab (anti-PD-1) caused 73–90% grades 3 and 4 irAEs.^20,21^ The high irAE rates impose an unacceptable burden to cancer patients and thus limited the clinical dosing. However, the mechanism of irAE remains largely unclear, which limits new approaches to overcome irAE. Since Ipilimumab does not block B7-CTLA-4 interactions if CTLA-4 is either expressed on cell surface or immobilized,^29^ irAE is not merely due to blocking B7-CTLA-4 interactions. However, since CTLA-4 mutations in mouse and human induce autoinflammatory diseases, it is plausible that CTLA-4 downregulation by anti-CTLA-4 antibody may have contributed to its irAE. Consistent with this notion, we demonstrated that two irAE-prone antibodies cause CTLA-4 downregulation, while two non-irAE-prone antibodies failed to do so. A stringent test for the hypothesis emerged only after our systemic understanding of the molecular mechanism of anti-CTLA-4 antibody-induced CTLA-4 downregulation.

CTLA-4 is a membrane protein that constitutively undergoes endocytosis regardless of B7 binding.^35^ In normal conditions, some internalized CTLA-4 molecules are recycled back to the plasma membrane while others are degraded in the lysosomal compartments. CTLA-4 recycling requires its interaction with an endosomal protein LRBA. Previous investigations demonstrated that LRBA plays an essential role in protecting CTLA-4 from being sorted to lysosomes for degradation, and promoting CTLA-4 recycling to the cell surface.^36^ Since patients with a LRBA mutation develop autoimmune diseases,^36^ we evaluated if irAE-prone anti-CTLA-4 antibodies interfere with CTLA-4 recycling. We have obtained several lines of evidence supporting this notion.

First, irAE-prone antibodies Ipilimumab and TremeIgG1 are targeted to lysosomes after endocytosis. Since their binding to CTLA-4 was unaffected by low pH found in late endosome and lysosome, it is predicted that CTLA-4 are also targeted to lysosome for degradation. This is confirmed by the data that showed lysosomal targeting by Ipilimumab and TremeIgG1 of the OFP-tagged CTLA-4.

Second, there is very little recycling of CTLA-4 molecules in Ipilimumab- and Tremelimumab-treated cells as inhibiting recycling does not appreciably increase cell surface CTLA-4.

Third, downregulation of CTLA-4 by Ipilimumab is prevented by an inhibitor of lysosomal degradation but not by proteasome inhibitor, affirming the role of antibody in inducing lysosomal degradation.

Conversely, anti-CTLA-4 mAbs that lose binding to CTLA-4 at low pH, such as HL12 or HL32, release CTLA-4 during antibody-induced internalization. Freed CTLA-4 recycles back to the cell surface, where it is required for Treg to protect mice against autoimmune diseases.^4^ The recycling requires CTLA-4-LRBA interactions as it is abrogated by a mutation of CTLA-4 that disrupts the interaction.^36^ The significance of intact CTLA-4 recycling in protecting against irAE is demonstrated by the fact that Primaquine, which blocks CTLA-4 recycling, confers irAE activity to HL12.

Since the Y201V mutation interferes with ligand-independent endocytosis of CTLA-4,^44^ it is of interest whether such an effect somehow contributed to defective recycling after treatment with pH-sensitive antibodies. We do not consider this is likely as our data demonstrated that in the presence of anti-CTLA-4 antibodies, endocytosis of the Y201V mutant is restored.

Taken together, our data demonstrate that differential pH sensitivity of anti-CTLA-4 antibodies dictates their effect on CTLA-4 recycling and irAE in a humanized mouse model that fully recapitulates human irAEs.

### A converging pathway for safer and more effective anti-CTLA-4 antibodies

The traditional concept of checkpoint blockade stipulates that anti-CTLA-4 antibodies promote tumor immunity by antagonizing a physiological checkpoint against autoimmunity.^27,28^ Given the fact that homozygous or heterozygous inactivation of the *CTLA4* gene^5–7^ or another gene involved in CTLA-4 recycling causes autoimmune diseases,^36^ effective antagonism of endogenous CTLA-4 function will necessarily increase the risk of autoimmune diseases. However, since recent studies have suggested that the immunotherapeutic effect of anti-CTLA-4 antibodies are independent of checkpoint blockade,^29^ CTLA-4-targeting cancer immunotherapy does not necessarily increase the risk of autoimmune diseases as it does not have to inactivate physiological function of CTLA-4. Surprisingly, a converging pathway for drug development arising from this study not only reduces toxicity of anti-CTLA-4 antibodies, but also increases their cancer therapeutic effect.

As discussed above, the key to a non-irAE-prone anti-CTLA-4 antibody is dissociation from CTLA-4 under low pH to allow its escape from lysosomal degradation and recycle to cell surface. Given the essential role of Treg depletion in the TME for CITE,^45^ it is of great interest to consider how the new approach of achieving less irAE would affect CITE. We have provided several lines of evidence for the notion that a pH-sensitive antibody is not only safer but also more effective in Treg depletion and tumor rejection than the pH-insensitive antibodies.

First, by preserving CTLA-4 on the cell surface, pH-sensitive antibodies leave higher ligand density for better ADCC. We have conclusively demonstrated that pH-sensitive antibodies exhibit better ADCC activity than the pH-insensitive antibodies. Since the enhanced ADCC activity is largely ablated when CTLA-4 lost the ability to interact with LRBA, a critical mediator for CTLA-4 recycling, preserving CTLA-4 recycling is an essential attribute to the better therapeutic effect of pH-sensitive antibody.

Second, pH-sensitive antibodies are more efficient in Treg depletion in TME. Thus, while both pH-sensitive and insensitive antibodies deplete Treg after repeated dosing^29^ or after 4 days of single dosing (this study), pH-sensitive antibodies are uniquely capable of depleting Treg in the TME within 24 h of administration.

Third, pH-sensitive antibodies exhibit better bioavailability. We have demonstrated that while pH-insensitive antibodies are targeted to the lysosome, the pH-sensitive antibodies translocate into Rab11^+^ recycling vehicles. It is well established that once an antibody enters a cell, its fate is largely dependent on whether it is bound to an antigen: antigen-free antibodies are rescued from degradation by its association with FcRn, while antigen-bound antibodies are targeted to lysosomal degradation.^46^ Since pH-sensitive anti-CTLA-4 antibodies disassociate from CTLA-4 in the endosome, they would be expected to be recycled rather than degraded, resulting in better bioavailability as we have demonstrated here.

Fourth, pH-sensitive antibodies are significantly more potent in inducing rejection of large tumors. We have reported that pH-sensitive and insensitive antibodies are both capable of inducing rejection of small tumors.^29,33^ Here we showed pH-sensitive antibodies are significantly more potent in causing rejection of large-established tumors, with two-fold higher complete tumor rejection rates. Large established tumors are significantly more resistant to immune rejection due to a well-established tumor microenvironment, one which has a very high percentage of Tregs. As such, it is not entirely unexpected that a better Treg-depleting antibody would be more potent in rejecting large tumors.

Clinical experience of Ipilimumab and Tremelimumab is consistent with a role for intratumorial Treg depletion for CITE of anti-CTLA-4 antibodies, although the relative weak ADCC activity and therapeutic efficacy of the clinical antibodies limit their power in testing the contribution of Treg depletion in a clinical response. Thus, in metastatic melanoma patients, Treg depletion in the TME correlates with the clinical response.^41^ In another study, neither Tremelimumab nor Ipilimumab reduced the absolute numbers of Tregs. However, Ipilimumab-treated melanoma had lower ratios of Treg/CD4 T cells than the untreated, while Tremelimumab-treated patients had higher ratios of Treg/CD4 T cells.^47^ The lack of Treg depletion by Tremelimumab is expected as Tremelimumab is an IgG2 isotype, which is largely devoid of ADCC activity. Although no head-to-head comparison had been reported comparing the clinical efficacy of the two antibodies, Tremelimumab has not reached clinical efficacy endpoint in phase III clinical trials.^37^ It would be of great interest to see if a more effective Ipilimumab variant can be obtained by antibody engineering at either the antigen binding domain, as detailed here for Tremelimumab, or at the Fc by well-established methods.^48^

Given the large body of evidence of the negative impact of intratumorial Treg on cancer prognosis, it is understandable that a more effective Treg-depleting antibody would promote tumor rejection. However, it is worth discussing how a more effective antibody would not negatively impact antibody safety as Treg have been shown to protect host against autoimmune diseases. This paradox is reconciled by several studies including ours, which clearly demonstrate that anti-CTLA-4 antibodies, regardless of their efficacy in Treg depletion in TME, do not reduce peripheral Treg numbers.^29–32^ The selective Treg depletion in TME is explained by higher density of CTLA-4 in Treg in TME.^29–32^ We showed that effector T cells in the tumor, which had lower levels of CTLA-4 than intratumor Treg, but similar CTLA-4 levels to Treg outside the tumor, were spared from depletion by anti-CTLA-4 antibodies.^29^ These data would argue higher levels of CTLA-4 as the primary mechanism for selective Treg depletion in the tumor, although more abundant ADCC/ADCP effectors in the tumor cannot be ruled out. The selective depletion of Treg in the tumors, but not those outside tumors, allowed a Treg-depleting antibody to avoid irAEs, providing that they do not cause CTLA-4 downregulation, which functionally inactivates Treg. The pH-sensitive anti-CTLA-4 antibodies described herein not only exhibit better Treg depletion in the tumor but also avoid irAE by preserving CTLA-4 levels to prevent irAE. In contrast, clinically used anti-CTLA-4 antibodies are largely pH-insensitive and rapidly induce lysosomal degradation of CTLA-4. Since heterozygous mutations of either *CTLA4* or *LRBA* are sufficient to cause autoimmune diseases in humans, quantitative downregulation of CTLA-4 is sufficient to cause autoimmune diseases and thus provide a straightforward explanation for toxicity of clinical antibodies.

Taken together, our data reveal a converging pathway for safer and more effective anti-CTLA-4 antibodies in cancer immunotherapy, thus sparing us the dilemma of having to choose between higher efficacy and less toxicity.

### Meeting the unmet medical needs in cancer immunotherapy by engineering pH-sensitive antibodies

Our data presented herein not only establish a fundamental converging pathway to improve safety and to enhance efficacy of anti-CTLA-4 antibodies, but also demonstrate how this new pathway can be explored to convert an irAE-prone and ineffective anti-CTLA-4 antibody into a safer and more effective one to meet the unmet medical needs in cancer immunotherapy. Our data suggest that this is achievable by engineering a pH-sensitive anti-CTLA-4 antibody.

By comparing two antibodies with only 2 or 3 Y > H mutations in the CDR3 region for their bioavailability, CTLA-4 downregulation, Treg depletion and rejection of large tumors, we have obtained compelling data that, by improving antibody bioavailability and preserving cell surface CTLA-4 on Treg, pH-sensitive antibodies confer better Treg depletion and more effective rejection of large established tumors. As a strong confirmation for converging features of safer and more effective antibodies, the pH-sensitive mutants also exhibit better safety. Thus, in addition to its low efficacy in causing rejection of large established tumors, TremeIgG1 was extremely toxic when used in combination with anti-PD-1, as it causes 93% lethality in young *Ctla4*^*h/h*^ KI mice. Two Y > H mutations in Ab157 not only yielded a more effective anti-CTLA-4 antibody, but also significantly improved its safety with only 15% mortality induced by Ab157 in combination therapy. Therefore, improved safety and efficacy can be achieved simply by engineering pH sensitivity into anti-CTLA-4 antibodies. However, although Ab157 is more pH-sensitive than HL12 and HL32, HL12 and HL32 remained less irAE-prone than Ab157. It is likely that HL12 and HL32, which was derived from an antibody identified through in vivo screening based on autoimmunity and immunotherapeutic efficacy, may have other features that needs to be explored for optimal CTLA-4 antibodies.

In considering pH-sensitive antibodies, it is of interest to consider low pH in tumor microenvironment. A recent study demonstrated that the pH at the surfaces of highly metastatic cells within tumors is about 6.1–6.4, and pH 6.7–6.9 in nonmetastatic tumors.^49^ This feature suggests that excessive pH sensitivity may prevent effective binding of anti-CTLA-4 antibodies in the tumor microenvironment.

Since the irAE-prone antibodies tend to activated T cells,^33^ they should increase biosynthesis of the CTLA-4, which, in the absence of circulating antibodies should result in restoration of CTLA-4 on Treg. It is of interest whether such a restoration will be sufficient to control irAE once they have developed. The impact of TremeIgG1 (treatment received between days 10-19) on long-term survival suggests that simply let antibodies decay is not sufficient to cure irAE.

Taken together, our data presented herein demonstrate that antibody-triggered lysosomal degradation of CTLA-4 not only causes toxicity, but also restrains anti-cancer immunity. This new paradigm enables the design or identification of safer and more effective anti-CTLA-4 antibodies for cancer immunotherapy. This outcome would not be possible by current approach that aims at breaking a physiological CTLA-4 checkpoint of immune tolerance to achieve better cancer immunity.

## Materials and methods

### Experimental animals

C57BL/6 mice that express the CTLA-4 protein with 100% identity to human CTLA-4 protein under the control of endogenous mouse *Ctla4* locus have been previously described.^34^ WT C57BL/6 mice were purchased from the Charles River Laboratories. All mice were maintained at the Research Animal Facility of Children’s Research Institute at the Children’s National Medical Center or the Institute of Human Virology at the University of Maryland Baltimore School of Medicine. All studies involving mice have been approved by the Institutional Animal Care and Use Committee.

### Antibodies

All anti-CTLA-4 antibodies used in this study are of IgG1 isotype. Two clones of humanized anti-CTLA-4 mAbs, HL12 and HL32 have been previously described.^29,33^ Recombinant Ipilimumab with amino acid sequence disclosed in WC500109302 and http://www.drugbank.ca/drugs/DB06186 was provided by Lakepharma Inc. (San Francisco, CA, USA). Clinical Ipilimumab was also used for much of the studies. Recombinant WT TremeIgG1 and mutated TremeIgG1 (Ab154-Ab159), as well as HL12 and HL32 were produced by Sydlabs Inc. (Boston, MA). Azide-free human IgG-Fc was purchased from Athens Research and Technology (Athens, GA, USA). Anti-mouse CD16/32 mAb 2.4G2 was purchased from Bio-X-Cell Inc. (West Lebanon, NH, USA). Biotinylation was completed by conjugating EZ-Link Sulfo-NHS-LC-Biotin (Thermo Fisher Scientific) to desired proteins/cells according to the manufacturer’s instructions. Alexa Fluor 488-conjugated goat anti-human IgG (H+L) cross-adsorbed secondary antibody and Alex Fluor 594-conjugated streptavidin were purchased from Thermo Fisher Scientific, USA.

### Primary human immune cells

Primary peripheral blood mononuclear cells were isolated from peripheral blood of healthy donors from the Children’s National Medical Center. The blood samples were negative for antibodies against hepatitis C virus, hepatitis B virus, HIV, and syphilis. All related procedures were performed with the approval of the Internal Review and Ethics Boards of the Children’s National Medical Center.

### Cell culture and treatment

CHO cells or HEK293T cells (ATCC® CRL-11268™) were transfected with WT OFP-hCTLA-4 (Sino Biological Inc.), OFP-hCTLA-Y201V (generated from OFP-hCTLA-4), WT GFP-hCTLA-4 or GFP-Y201V-hCTLA-4 (gifts by Dr. Michael lenardo) by Lipofectamine 3000 (Cat# L3000015, Thermo Fisher). Cells that were stably transfected with CTLA-4 have been reported.^29^ The murine colon tumor cell line MC38 was described previously.^29^ All cell lines were incubated at 37 °C and were maintained in an atmosphere containing 5% CO_2_. Cells were grown in DMEM (Dulbecco’s Modified Eagle Medium, Gibco) supplemented with 10% FBS (Hyclone), 100 units/mL of penicillin and 100 μg/mL of streptomycin (Gibco). The ADCC effector Jurkat T cells were cultured in RPMI 1640 medium supplemented with 10% FBS, 100 μg/mL hygromycin B, 250 μg/mL G-418 sulfate solution, 1 mM sodium pyruvate and 0.1 mM MEM nonessential amino acids.

Human PBMCs were isolated by Ficoll density gradient centrifugation and were activated by the stimulation with 1 μg/mL of anti-CD3 (OKT3, Invitrogen) and anti-CD28 (CD28.2, Biolegend) for 2 days. Surface and intracellular CTLA-4 were analyzed by flow cytometry and confocal microscopy. Unstimulated or activated human PBMCs were cultured in RPMI Medium 1640 (Gibco) supplemented with 10% FBS (Hyclone), 100 units/mL of penicillin and 100 μg/mL of streptomycin (Gibco).

### pH-dependent antigen binding of anti-CTLA-4 mAbs

His-hCTLA-4 (0.5 μg/mL) was coated in ELISA plates and different anti-CTLA-4 mAbs were added at 10 μg/mL or 1 μg/mL in the buffer at different pH range from pH 4.0 to 7.0, for 3 h at room temperature. Antibodies binding with CTLA-4 were measured by using horse-radish perioxidase-labeled anti-human IgG antibodies (Pierce High Sensitivity NeutrAvidin-HRP, Thermo Scientific Inc.). To mimic the in vivo status, different anti-CTLA-4 mAbs were added at 10 μg/mL or 1 μg/mL at pH 7.0 at room temperature for 1.5 h. After extra antibodies were washed away, binding of CTLA-4 was detected followed by 1.5 h incubation at lower pH buffer (pH 4.5, 5.5, and 6). Proteins were coated in bicarbonate buffer (0.1 M) at 4 °C overnight and the binding assays were performed at room temperature.

### Generation of Tremelimumab IgG1 variants with pH-dependent binding

TremeIgG1 variants with histidine mutation in CDR residues were generated by site-directed mutagenesis. ELISA or SPR measurements of histidine-mutated variants were performed to evaluate their ability to bind to CTLA-4 pH-dependently.

### Plasma membrane protein isolation

Plasma membrane proteins of treated cells were isolated by using MINUTE^TM^ plasma membrane protein isolation and cell fractionation kit (SM-005, Invent Biotechnologies, INC.). In brief, cells are first sensitized by buffer A before passing through the proprietary filter in a zigzag manner when high-speed centrifugal force is applied, resulting in a cell lysate containing ruptured cell membranes and intact nuclei. As a result, the nuclear contaminations are virtually eliminated. Plasma membrane is further separated from the cell lysate (a mixture of crude membranes, intact nuclei, cytosol proteins and organelles) by subsequent differential and density centrifugation. The final fraction of plasma membrane was identified by testing plasma membrane marker, Na^+^/K^+^ ATPase alpha1, by western blotting.

### Antibody competition assay

Human primary PBMCs were treated with/without 10 μg/mL of hIgG, Ipilimumab, TremeIgG1, HL12 and HL32 at 4 °C for 30 min. After washing away the unbound antibodies, cells were stained with commercial mouse anti-human monoclonal antibody (clone: BNI3). Surface level of CTLA-4 effected by the treated anti-CTLA-4 mAbs were compared by flow cytometry (Supplementary Information, Fig. S2f).

### Flow cytometry

To exclude the competition between the treated anti-CTLA-4 mAbs with the staining antibody of CTLA-4 (Cat# 369604, Biolegend, Clone: BNI3), all the CTLA-4-positive cells treated with anti-CTLA-4 mAbs in vitro or in vivo, were incubated with their treated anti-CTLA-4 mAbs (10 μg/mL) for 30 min at 4 °C before the anti-CTLA-4 staining with BNI3 clone. The isotype control (Cat# 400214, Biolegend) was used for all the BNI3 staining.

Cells were stained with fluorochrome-conjugated monoclonal antibodies against: human CD8 (Cat# 560662, clone: RPA-T8, BD Pharmingen), human CD4(Cat# 344604, Biolegend), human CD45 (Cat# 304022, Biolegend), human CD25 (Cat# 356122, Biolegend), human Fxop3 (Cat# 17-4777-42, eBioscience), mouse CD8 (Cat# 45-0081-82, eBioscience), mouse CD4 (Cat# 100531, Biolegend), mouse CD45 (Cat# 47-0451-82, eBioscience), mouse CD25 (Cat# 530-251-82, eBioscience) and mouse Foxp3 (Cat# 17-5773-82, eBioscience). Intracellular staining was performed with Intracellular Fixation and Permeabilization kit (Cat# 88-8824, eBioscience) according to the manufacturer’s instructions. The samples were analyzed by the BD Canton II Flow cytometer and data were analyzed by Flowjo software. Total CTLA-4 levels were determined by fixing and permeabilizing the cells, staining for CTLA-4 and Foxp3, then analysis by flow cytometry. The level of CTLA-4 was shown as the geometric mean of the fluorescence.

### Western blot

Cells were collected and lysed in M2 buffer (20 mM Tris, pH 7, 0.5% NP40, 250 mM NaCl, 3 mM EDTA, 3 mM EGTA) with proteinase inhibitor cocktail (Cat# 78446, Thermo Fisher). Cell lysates were separated by 4%-12% SDS–PAGE and analyzed by immunoblotting. The antibodies used: anti-CTLA-4 (Cat# clone: H126, sc-9094, Santa Cruz 1:1 000), anti-Na^+^ K^+^ ATPase (Cat# ab7671, Abcam), anti-Tubulin (Cat# T9026, Sigma) and anti-β-actin (ACTB) (Cat# A2228, Sigma). For LRBA, cell lysates were separated using 3%–8% Tris-acetate gels (Invitrogen), transferred onto nitrocellulose, and immunoblotted with primary antibodies to LRBA (Cat# HPA023597, Sigma), CTLA-4 (Santa Cruz) and β-actin. The proteins were visualized by chemiluminescence (ECL; Thermo Fisher). The quantitation of CTLA-4 were normalized to control proteins ACTB or Na^+^-K^+^ ATPase (untreated or hIgG groups shown as 1 in the Figures).

### Co-immunoprecipitation

CTLA-4-positive cells were lysed by incubation for 30 min at 4 °C with M2 buffer with proteinase inhibitor cocktail (Cat# 78446, Thermo Fisher). After a centrifugation at 14000 × g for 15 min at 4 °C, the supernatant was prepared to perform immunoprecipitation and incubated with Protein G Agarose (Cat# 11243233001, Sigma-Aldrich) and rabbit anti-GFP antibody (Cat# ab290, Abcam; 1:500) for overnight at 4 °C. Protein G Agarose beads-antigen–antibody complex was collected after a centrifugation at 14,000 rpm for 5 s and washed by M2 lysis buffer five times. The complex was suspended with 60 μL 5 × simple buffer and cooked for 10 min, followed by analyzing the supernatant by SDS–PAGE and immunoblotting.

### Immunofluorescence

Ipilimumab, TremeIgG1, and its variants or HL12, HL32 were labeled with AF488 by Alexa Fluor™ 488 Antibody Labeling Kit (A20181, Thermo Fisher) according to the manufacturer’s instructions. CHO stable cell lines expressing hCTLA-4 were treated with labeled antibodies at 4 °C and transferred to 37 °C after extra antibodies were washed away. Cells were further stained with lysotracker (far red for most of the experiments, while Red DND-99 for human Tregs that need to be fixed for confocal). Endocytosis of the surface CTLA-4 and the co-localization between surface CTLA-4 and lysosomes were acquired on a ZEISS LSM 800 confocal microscope.

CHO cells transfected with human ds-Red-Rab11 as well as activated human Peripheral blood lymphocytes (PBL) were incubated with either Ipilimumab-AF488 or HL12-AF488. HEK293T cells transfected with hCTLA-4-GFP or CTLA-4-Y-201V was incubated with control IgG, Ipilimumab, HL12 or TremeIgG1. Fluoresce images of these cells were taken by ZEISS LSM 800 confocal microscope.

### Image acquisition: confocal microscopy

Confocal images of cell surface and cell cytosol-associated fluorescence were acquired using the Zeiss LSM 800 confocal system and Zen Blue software (Carl Zeiss Microscopy, Germany). Four laser lines, 405 nm (blue, for nuclei), 488 nm (green, for mAbs or CTLA-4), 594 nm (for OPF-CTLA-4 or for CTLA-4 or Biotin-labeled surface proteins) and 631 nm (far red, for lysotracker) were used in our imaging experiments. Blue, green and far red signals were separated by a quad DAPI/FITC/TRITC/Cy5 dichroic beam splitter. These images were further acquired using a Gasp detector (Carl Zeiss LSM 800, Germany). A Plan-Apochromat 63 × /1.4 Oil DIC objective (Carl Zeiss LSM 800, Germany) was used to visualize multi-colored cell samples. All the parameters used in confocal microscopy were consistent in each experiment, including the laser excitation power, detector and offset gain. In addition, signal to noise ratio was secured by averaging data for each imaging field acquired. The saturated signal was avoided by using the range indicator in all experiments. The confocal system is equipped with the incubation unit for the CO_2_/ T control (Tokai, Japan).

### ADCC reporter assay

ADCC bioassay effector cells in propagation model (Catalog number G7102) with engineered expressions of FcγRIIIA (V158) and an NFAT (nuclear factor of activated T cells)-dependent firefly luciferase reporter gene were purchased from Promega Corporation (Madison, WI, USA). 293T cells with stable expression of OFP-tagged human CTLA-4 were used as the targeted cells. The in vitro ADCC assay was performed following the instructions from the manufacturer of ADCC effector cells (Promega). Briefly, the effector cells (2 × 10^4^ cells/well) were co-cultured with target 293T cells at an effector-to-target ratio of 1:1, and along with indicated concentrations of anti-human CTLA-4 antibodies or control antibody in RPMI medium supplemented with 4% low-IgG serum in white, flat-bottom 96-well assay plates (Corning™). After incubation of plates at 37 °C for 20 h, 50 µL Bio-Glo™ luciferase assay reagent (Promega) was added into each well and the luminescence intensity was measured with a plate reader (SpectraMax iD3 Multi-Mode Microplate Readers, Molecular Devices, LLC, San Jose, CA, USA).

### Surface plasmon resonance (SPR) measurement of anti-CTLA-4 mAbs affinity

TremeIgG1 and its variants were assessed for their binding affinity to his-CTLA-4 by SPR analysis using a Biacore T100 biosensor (GE Healthcare). 1000 response units (RU) of Protein A from Staphlococcus aureus (Sigma-Aldrich) were immobilized on flow cells 1 and 2 of a Series S Sensor Chip CM5 (GE, BR100530). Approximately 150 RUs of antibody were directly captured on flow cell 2. Binding experiments were carried out in 10 mM HEPES, 150 mM NaCl, 0.05% (v/v) Tween20 with pH 7.0 at 25 °C. A twofold titration series of his-CTLA-4 (0.976 nM-500 nM) were used. In between runs, the sensor surface was regenerated with two 45 s injections of 20 mM HCl. Sensorgrams were double referenced against the control flow cell (Fc1) and three buffer injections.

### irAE model and histopathology analysis

Young *Ctla4*^*h/h*^ mice were treated, respectively, with indicated antibodies at a dose of 100 μg/mouse/injection every 3 days for total four injections, starting on day 10 of birth. To avoid cage variation, mice in the same cages were individually tagged and treated with different antibodies. To avoid gender and weight variation, female mice with similar weight (4.5–5.3 g) were used for all the study, although similar trends were observed in male mice. For Kaplan–Meier survival analyses, mice are considered to have reached endpoint if they become moribund or died. The experiments were performed double blind.

Organs from mice that received therapeutic or control antibodies were harvested and fixed in formalin. Hematoxylin and eosin (H&E) staining was performed by Histoserv, Inc. Inflammation status of those organs were scored double blind. Score criteria has been described previously.^33^ Data shown are the scores for single organs or the combined scores of all organs examined.

### Complete blood counts

Blood samples (50 μL) were collected when mice were 41 days old by using tubes with K2EDTA (BD) and analyzed by HEMAVET HV950 (Drew Scientific Group, Miami Lakes, FL, USA) following the manufacture’s manual.

### Tumor growth measurements

*Ctla4*^*h/h*^ mice were challenged with given the colorectal cancer cell MC38 by 0.5 million per mouse. Immunotherapies were initiated at 7–17 days after injection of tumor cells with indicated doses. The tumor volumes calculated by the formula: volume = width^2^ × length/2 were recorded every 3 days. The tumor diameters were calculated by the formula:

$${\mathrm{Diameter}} = \sqrt {\left( {{\mathrm{long}}\,{\mathrm{diameter}} \times {\mathrm{short}}\,{\mathrm{diameter}}} \right)}$$

### Isolation of mononuclear cells in lung and tumor

Lung or tumor was minced into small (1 to 2 mm) pieces and digested with 1 mg/mL collagenase IV (Sigma-Aldrich, cat# C5138), 0.1 mg/mL Hyaluronidase (Sigma-Aldrich, cat# H6254) and 0.01 mg/mL deoxyribonuclease I (Sigma-Aldrich, cat# D5025). The cells were sequentially filtered through 100 µm cell strainer. The cells were then centrifuged at 2500 rpm for 5 min. Mononuclear cells were collected after one time wash with PBS. All isolated mononuclear cells were analyzed by flow cytometry.

### Statistical analysis

For each statistical analysis, appropriate tests were selected on the basis of whether the data with outlier deletion was normally distributed by using the D’Agostino & Pearson normality test. Data were analyzed using an unpaired two-tailed Student’s *t* test to compare between two groups, one-way analysis of variance (ANOVA) or Kruskal–Wallis test for multiple comparisons. No samples were excluded from the analysis, and experiments were not randomized except where specified. Blinding was not done during animal group allocation but was done for some measurements made in the study (i.e., scoring of histology). In the graphs, y-axis error bars represent S.E.M. or S.D. as indicated. Statistical calculations were performed using Excel (Microsoft), GraphPad Prism software (GraphPad Software, San Diego, California). *p < 0.05, **p < 0.01, ***p < 0.001, ****p < 0.0001.

## Supplementary information

Supplementary information, Figure S1

Supplementary information, Figure S2

Supplementary information, Figure S3

Supplementary information, Figure S4

Supplementary information, Figure S5

Supplementary information, Table S1
