# Supplementary material for: Hijacking antibody-induced CTLA-4 lysosomal degradation for safer and more effective cancer immunotherapy
Source: Cell Res. 2019 Jul 2;29(8):609–27. doi: 10.1038/s41422-019-0184-1 (PMC6796842; doi:10.1038/s41422-019-0184-1)
Supplement: Supplementary file 1 — Supplementary information, Figure S1 [file 41422_2019_184_MOESM1_ESM.pdf]

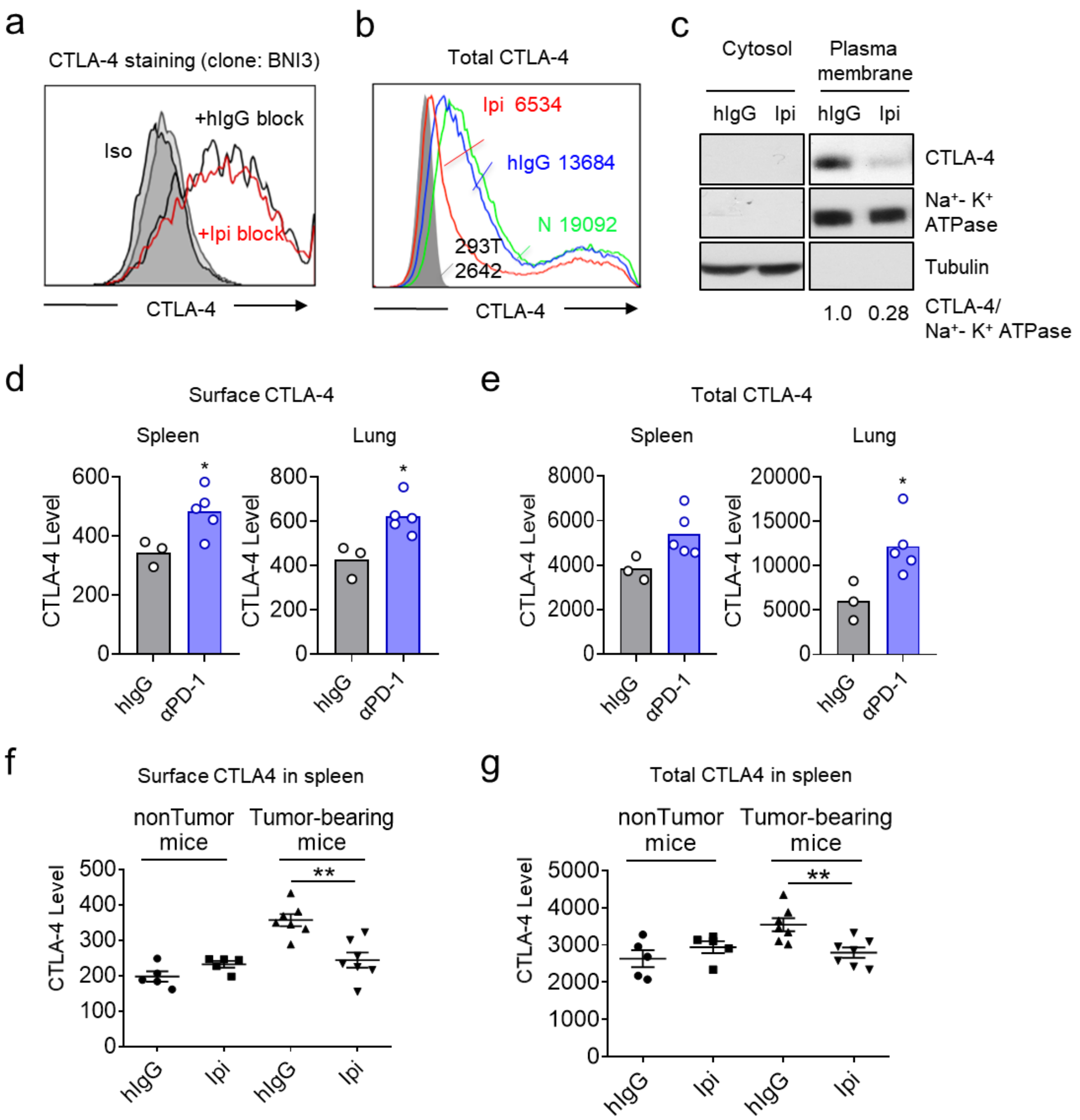

Figure S1. Ipilimumab markedly down-regulates the level of cell surface CTLA-4, Related to Figure 1

(a) Mouse splenocytes were isolated and stained with surface markers (CD45, CD4 and CD8). Cells were fixed and incubated with 10 μg/ml of hIgG (hIgG block) or Ipilimumab (Ipi block) at 4ºC for half an hour. After several washing, cells were staining with anti-

CTLA-4 (BNI3) and anti-Foxp3. The CTLA-4 expression in Foxp3$^+$ Tregs were shown. A color-matched Isotype control staining was performed for BNI3 staining (shown as Iso). (b) HEK293T cells transfected with OFP-tagged human CTLA-4 were treated with either control hIgG or Ipilimumab for 4 hrs at 37°C. The fluorescence of OFP was detected by flow cytometry and the geometric mean of the fluorescence were shown. (c) Plasma membrane proteins in CTLA-4 transfected HEK293T cells were isolated. CTLA-4, Na^+-K^+ ATPase and Tubulin were detected by western blot. (d&e) CTLA-4$^{h/h}$-KI neonatal mice were i.p. treated with 100 µg of control hIgG Fc or mouse anti-PD-1 antibody. After 24 hrs, cell surface (d) and total CTLA-4 (e) in Tregs isolated from mice lung and spleen were evaluated by flow cytometry. (f&g) naïve or MC38 bearing-*Ctla4*$^{h/h}$ mice (n=5-7) were i.p. treated with control hIgG Fc or Ipilimumab (100 μg/mouse) on day 17 after tumor inoculation (tumors had an average size of 8-10 mm in diameter). Spleen Treg from the *Ctla4*$^{h/h}$ adult mice were analyzed for cell surface (f) and total (g) CTLA-4 expression by flow cytometry. Data in d-g are mean ± SEM. *p<0.05, **p<0.01, ***p<0.001, ****p<0.0001. Representative data of two independent experiments in (a) were shown. Representative data of three independent experiments in (b) and (c) were shown. Representative data of three independent experiments in (d-g) were shown.
