# Supplementary material for: Hijacking antibody-induced CTLA-4 lysosomal degradation for safer and more effective cancer immunotherapy
Source: Cell Res. 2019 Jul 2;29(8):609–27. doi: 10.1038/s41422-019-0184-1 (PMC6796842; doi:10.1038/s41422-019-0184-1)
Supplement: Supplementary file 2 — Supplementary information, Figure S2 [file 41422_2019_184_MOESM2_ESM.pdf]

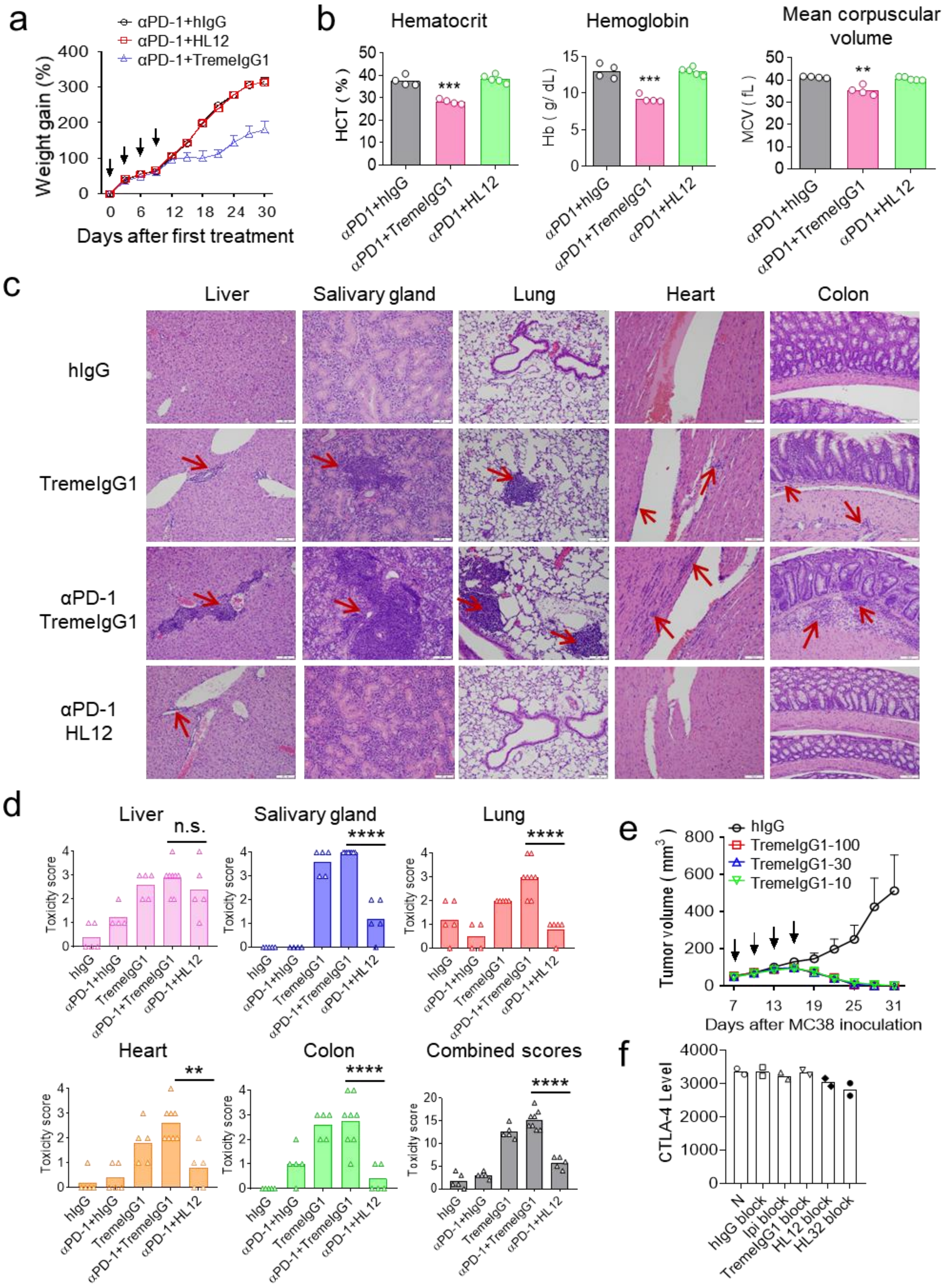

a
αPD-1+hIgG
αPD-1+HL12
αPD-1+TremeIgG1
Weight gain (%)
Days after first treatment
b
Hematocrit
Hemoglobin
Mean corpuscular volume
HCT (%)
Hb (g/ dL)
MCV (fL)
αPD1+hIgG
αPD1+TremeIgG1
αPD1+HL12
c
Liver
Salivary gland
Lung
Heart
Colon
hIgG
TremeIgG1
αPD-1 TremeIgG1
αPD-1 HL12
d
Liver
Salivary gland
Lung
Heart
Colon
Combined scores
Toxicity score
n.s.
hIgG
αPD-1+hIgG
TremeIgG1
αPD-1+TremeIgG1
αPD-1+HL12
e
hIgG
TremeIgG1-100
TremeIgG1-30
TremeIgG1-10
Tumor volume (mm^3)
Days after MC38 inoculation
f
CTLA-4 Level
N
hIgG block
Ipi block
TremeIgG1 block
HL12 block
HL32 block

Figure S2. Down-regulation of surface CTLA-4 by anti-CTLA-4 mAbs correlates with their irAE, Related to Figure 2

(a) C57BL/6 *CTLA4*$^{h/h}$ male mice were treated with control hIgG Fc plus anti-PD-1, TremeIgG1 plus anti-PD-1, or HL12 plus anti-PD-1 respectively, at a dose of 100 µg/mouse/injection on days 10, 13, 16 and 19 after birth. Means and SEM of the weight gains of mice receiving different treatments are shown. n=5. One mouse in the TremeIgG1 treated group died at day 31. (b) The CBC analysis of mice blood from (A) was performed on day 41 after birth. Data of blood hematocrit (HCT), total hemoglobin (Hb) and Mean Corpuscular Volume (MCV) are shown. (c) Representative images of H&E stained paraffin sections from different organs of mice with the indicated treatments. Representative inflammatory foci are marked with arrows. Scale bar, 200 µm. (d) Toxicity scores of internal organs and glands in (c). Data are mean ± S.E.M., n = 5–8 mice per group. Data were analyzed by one-way ANOVA with Bonferroni's multiple comparison tests. (e) MC38 bearing-*Ctla4*$^{h/h}$ female mice (n=5) were i.p. treated with either control hIgG Fc (100 µg) or TremeIgG1 (10 µg, 30 µg or 100 µg) on day 7, 10, 13 and 16 after tumor inoculation. (f) Antibody competition was analysis by adding saturated doses of anti-CTLA-4 mAbs to the cells before CTLA-4 staining with BNI3 when comparing with the hIgG group. Splenocytes of *Ctla4*$^{h/h}$ mice were used for this test. Data are mean ± SEM. *p<0.05, **p<0.01, ***p<0.001, ****p<0.0001. Representative data of three independent experiments in (a), (b) and (e) were shown. The samples in (c) and (d) were collected from three independent experiments and have been scored double-blind. Representative data of two independent experiments were shown in (f).
