# Supplementary material for: Hijacking antibody-induced CTLA-4 lysosomal degradation for safer and more effective cancer immunotherapy
Source: Cell Res. 2019 Jul 2;29(8):609–27. doi: 10.1038/s41422-019-0184-1 (PMC6796842; doi:10.1038/s41422-019-0184-1)
Supplement: Supplementary file 3 — Supplementary information, Figure S3 [file 41422_2019_184_MOESM3_ESM.pdf]

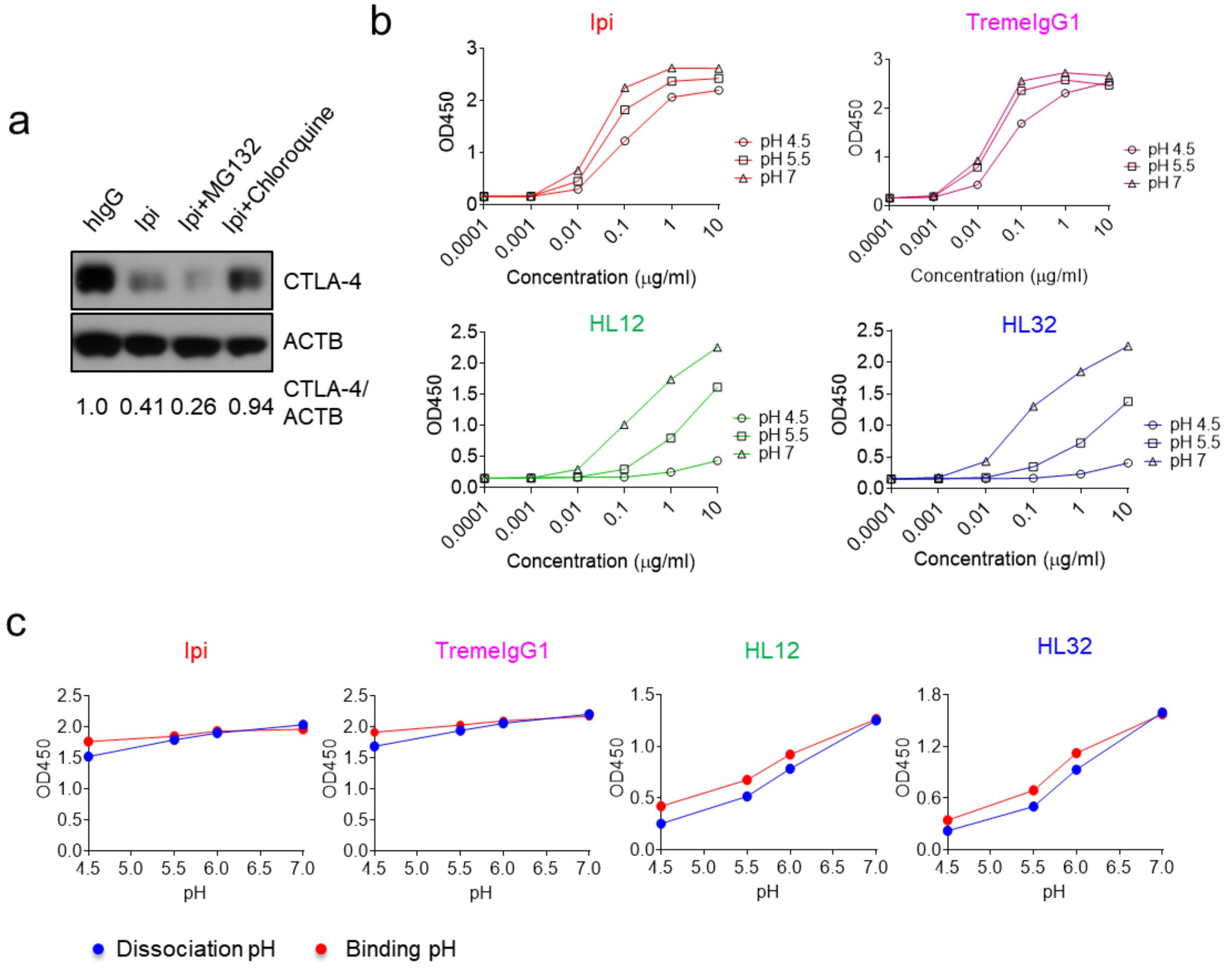

Figure S3. pH-insensitive target binding of irAE-prone anti-CTLA-4 mAbs triggers lysosomal degradation of CTLA-4, Related to Figure 3. (a) HEK293T cells transfected with hCTLA-4 were treated with either control hIgG Fc or Ipilimumab, with or without MG132 (1 µM) or chloroquine (50 µM), for 4hrs. The CTLA-4 protein levels were analyzed by Immunoblot. (b) His-hCTLA-4 (0.5 µg/ml) was coated onto ELISA plates and anti-CTLA4-mAbs with different dilutions were added in the buffer at pH 4.5, 5.5 and 7.0. Then antibodies binding with CTLA-4 were detected. (c) His-hCTLA-4 (0.5 µg/ml) was bound with different anti-CTLA4-mAbs at 10 µg/ml at pH 7.0. After extra antibodies were washed

away, binding of CTLA-4 was detected followed by 2hrs incubation at lower pH buffer (pH 4.5, 5.5, 6 and 7). Data are means of duplicate optical density at 450 nm. Representative data of two independent experiments are shown in (a), (b) and (c).
