# Supplementary material for: Hijacking antibody-induced CTLA-4 lysosomal degradation for safer and more effective cancer immunotherapy
Source: Cell Res. 2019 Jul 2;29(8):609–27. doi: 10.1038/s41422-019-0184-1 (PMC6796842; doi:10.1038/s41422-019-0184-1)
Supplement: Supplementary file 4 — Supplementary information, Figure S4 [file 41422_2019_184_MOESM4_ESM.pdf]

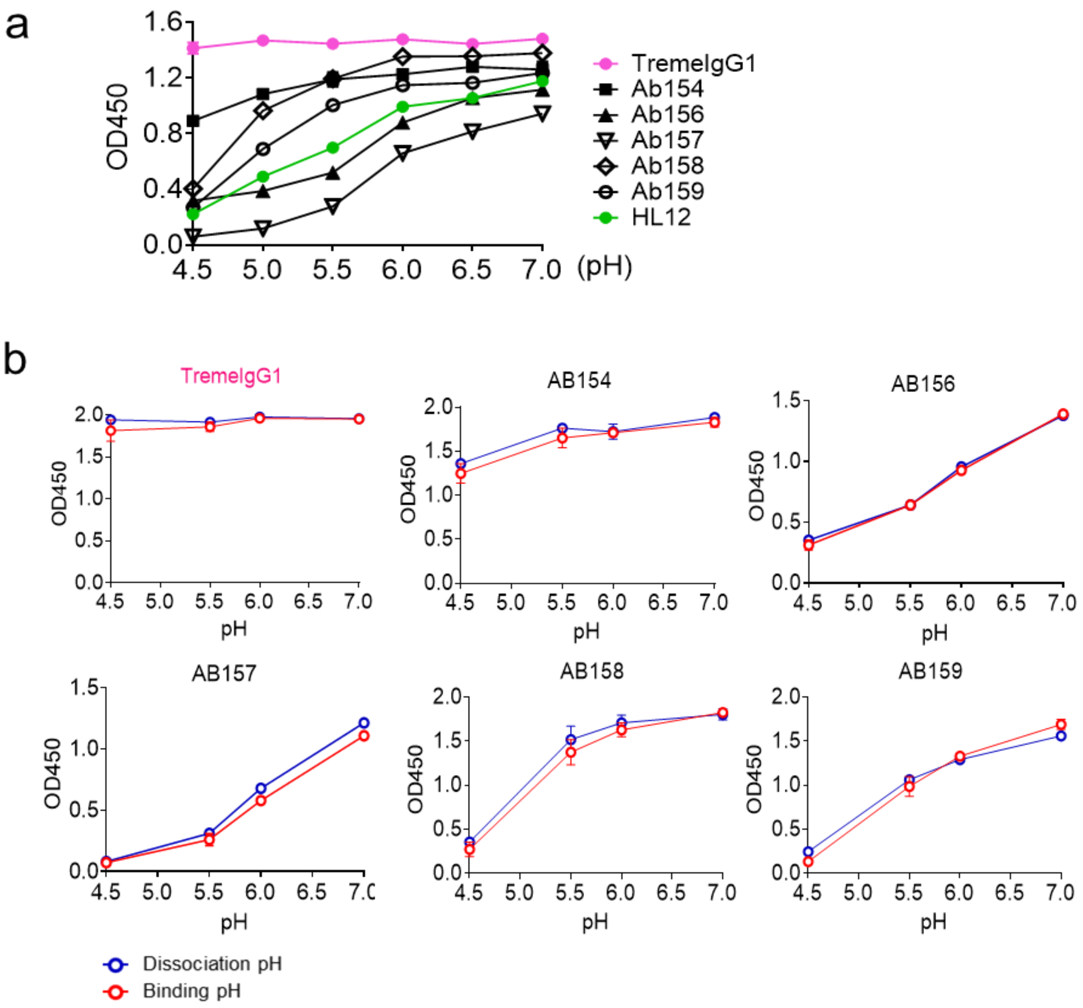

Figure S4. CTLA-4 recycling by engineered pH-dependent antibody binding reduces anti-CTLA-4-induced irAE, Related to Figure 7

(a) Original data of Figure 7A without normalization. (b) His-hCTLA-4 (0.5 μg/ml) was coated onto ELISA plates. TremeIgG1 and its variants were added at 1 μg/ml at pH 7.0. After extra antibodies were washed away, binding of CTLA-4 was detected followed by 2h incubation at lower pH buffer (pH 4.5, 5.5, 6 and 7). Data in are means of duplicate optical density at 450 nm. Representative data of two independent experiments are shown.
