# Supplementary material for: Hijacking antibody-induced CTLA-4 lysosomal degradation for safer and more effective cancer immunotherapy
Source: Cell Res. 2019 Jul 2;29(8):609–27. doi: 10.1038/s41422-019-0184-1 (PMC6796842; doi:10.1038/s41422-019-0184-1)
Supplement: Supplementary file 5 — Supplementary information, Figure S5 [file 41422_2019_184_MOESM5_ESM.pdf]

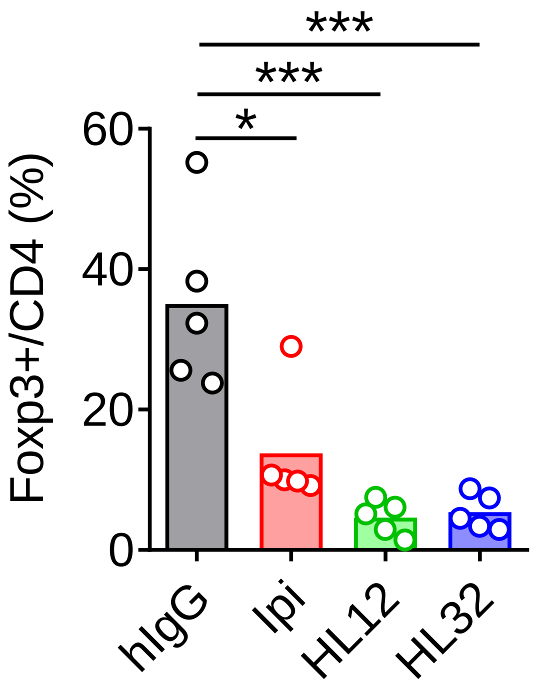

Figure S5. pH sensitivity confers improved therapeutic effect of anti-CTLA-4 antibodies, Related to Figure 8. MC38 bearing-*Ctla4*$^{h/h}$ mice (n=5) were treated with either control hIgG, Ipilimumab (Ipi), HL12 or HL32 (100 μg/mouse) on day 6 after tumor inoculation. Selective depletion of Treg cells in the tumor microenvironment was determined by % Treg cells among CD4 T cells at 96 hrs after antibody treatment. Data are mean ± SEM. *p<0.05, **p<0.01, ***p<0.001, ****p<0.0001. Representative data of two independent experiments are shown.
