# Supplementary material for: Hijacking antibody-induced CTLA-4 lysosomal degradation for safer and more effective cancer immunotherapy
Source: Cell Res. 2019 Jul 2;29(8):609–27. doi: 10.1038/s41422-019-0184-1 (PMC6796842; doi:10.1038/s41422-019-0184-1)
Supplement: Supplementary file 6 — Supplementary information, Table S1 [file 41422_2019_184_MOESM6_ESM.pdf]

SUPPLEMENTARY data

Table S1 Binding kinetics and affinity of pH-dependent binding variants of TremeIgG1

Antibody	k_a ($M^{-1}s^{-1}$)	k_d (s^{-1})	K_D (M)	Mutations	Location
TremeIgG1	5.271E + 05	7.729E - 05	1.466E - 10	N/A	N/A
Ab154	1.643E + 05	2.018E - 04	1.228E - 09	S31H, Y32H, W36H	CDR1, IgH
Ab155	No binding	No binding	No binding	S31H, Y32H, W36H<br>Y21H, D34H	CDR1, IgH<br>CDR1, IgK
Ab156	8.271E + 04	3.688E - 04	4.459E - 09	Y106H, Y107H, Y108H	CDR3, IgH
Ab157	1.112E + 05	2.583E - 04	2.322E - 09	Y107H, Y108H	CDR3, IgH
Ab158	3.047E + 05	1.653E - 04	5.425E - 10	Y107H	CDR3, IgH
Ab159	5.235E + 05	2.024E - 04	3.867E - 10	Y59H, Y60H	CDR2, IgH

Association rate (k_a), dissociation rate (k_d) and binding affinity (K_D) of TremeIgG1 and its pH dependent variants are shown. His mutations and related locations were listed.
